# Identification and Comparison of Chemosensory Genes in the Antennal Transcriptomes of *Eucryptorrhynchus scrobiculatus* and *E. brandti* Fed on *Ailanthus altissima*

**DOI:** 10.3389/fphys.2018.01652

**Published:** 2018-11-20

**Authors:** Xiaojian Wen, Qian Wang, Peng Gao, Junbao Wen

**Affiliations:** Beijing Key Laboratory for Forest Pests Control, College of Forestry, Beijing Forestry University, Beijing, China

**Keywords:** coexistence, *Eucryptorrhynchus scrobiculatus*, *Eucryptorrhynchus brandti*, *Ailanthus altissima*, chemosensory genes, olfactory differentiation

## Abstract

The key to the coexistence of two or more species on the same host is ecological niche separation. Adult *Eucryptorrhynchus scrobiculatus* and *E. brandti* both feed on the tree of heaven, *Ailanthus altissima*, but on different sections of the plant. Olfaction plays a vital role in foraging for food resources. Chemosensory genes on the antennae, the main organ for insect olfaction, might explain their feeding differentiation. In the present study, we identified 130 and 129 putative chemosensory genes in *E. scrobiculatus* and *E. brandti*, respectively, by antennal transcriptome sequencing, including 31 odorant-binding proteins (OBPs), 11 chemosensory proteins (CSPs), 49 odorant receptors (ORs), 17 ionotropic receptors (IRs), 19 gustatory receptors (GRs), and three sensory neuron membrane proteins (SNMPs) in *E. scrobiculatus* and 28 OBPs, 11 CSPs, 45 ORs, 25 IRs, 17 GRs, and three SNMPs in *E. brandti*. We inferred that *EscrOBP8* (*EscrPBP1*), *EscrOBP24* (*EscrPBP2*) and *EbraOBP8* (*EbraPBP1*), *EbraOBP24* (*EbraPBP2*) were putative PBPs by the phylogenetic analysis. We identified species-specific OR transcripts (10 EscrORs and 8 EbraORs) with potential roles in the recognition of specific volatiles of *A. altissima*. In addition to conserved “antennal IRs,” we also found several “divergent IRs” orthologues in *E. scrobiculatus* and *E. brandti*, such as *EscrIR16*, *EbraIR19*, and *EbraIR20*. Compared with other chemosensory genes, GRs between *E. scrobiculatus* and *E. brandti* shared lower amino acid identities, which could explain the different feeding habits of the species. We examined OBP expression patterns in various tissues and sexes. Although amino acid sequence similarities were high between EscrOBPs and EbraOBPs, the homologous OBPs showed different tissue expression pattern between two weevils. Our systematic comparison of chemosensory genes in *E. scrobiculatus* and *E. brandti* provides a foundation for studies of olfaction and olfactory differentiation in the two weevils as well as a theoretical basis for studying species differentiation.

## Introduction

Over a long period of evolution, phytophagous insects and their hosts have formed a complete system of co-evolution. For two or more species living on the same host plant with a similar niche, competition over food resources is inevitable; only differentiation in time, space, or nutrition can reduce interspecific competition and enable coexistence. Ecologists generally believe that niche separation usually occurs in order to achieve coexistence for species with similar niche, and niche separation is the key to species coexistence (Caldwell and Vitt, 1999; Sedio and Ostling, 2013). *Eucryptorrhynchus scrobiculatus* Motschulsky and *E. brandti* (Harold) (Coleoptera: Curculionidae) are sympatric, closely related species native to China and are highly host-specific, feeding on the tree of heaven, *Ailanthus altissima* (Mill.) Swingle, and its variant *A. altissima* var. Qiantouchun (Alonsozarazaga and Lyal, 1999; Yang et al., 2008; Herrick et al., 2012; Chao and Chen, 2015). The mixed cooccurrence of *E. scrobiculatus* and *E. brandti* results in extensive *A. altissima* deaths in the Ningxia Hui autonomous region (Hu et al., 2012; Yu et al., 2012). The coexistence of *E. scrobiculatus* and *E. brandti* can be explained by significant differentiation of trophic niches; *E. scrobiculatus* adults feed on 1-year-old branches, perennial branches, and petioles, while *E. brandti* adults feed on the stem of *A. altissima* (Ji et al., 2017). The role of olfaction in their feeding differentiation is unknown.

Olfaction plays a vital role in insect foraging for food resources (Leal, 2013). Antennae are the main olfactory organs in insects. Chemosensory genes in the antennae, such as genes encoding odorant-binding proteins (OBPs), chemosensory proteins (CSPs), odorant receptors (ORs), ionotropic receptors (IRs), gustatory receptors (GRs), sensory neuron membrane proteins (SNMPs), and odorant-degrading enzymes (ODEs), participate in the recognition of odor molecules (Vogt and Riddiford, 1981; Raming et al., 1993). A large number of experiments have shown that chemosensory genes in the antennae are involved in the chemical communication between insects and plants. For example, Swarup et al. (2011) found that responses to a specific odorant are frequently affected by the suppression of the expression of multiple OBPs by RNAi in *Drosophila melanogaster*. Wu et al. (2016) found that a reduction in *Bdor83a-2* transcript abundance leads to a decrease in neuronal and behavioral responses to selected attractants, suggesting that *Bdor83a-2* mediates behavioral responses to attractant semiochemicals. Previous studies have shown that gustation plays an important role in host selection (Hanson and Dethier, 1973; Boer and Hanson, 1987). GRs might be involved in gustatory (Jiang et al., 2015) and olfactory processes (Agnihotri et al., 2016).

Few studies have examined the mechanism of olfactory differentiation in closely related species; however, olfaction-related gene expression divergence is linked to differences in host preference between closely related species. Based on different blood feeding behaviors, Yan (2014) identified ORs with significant differences in expression between *Culex pipiens quinquefasciatus* and *Cx. molestus*, and found that *CquiOR5* is involved in the blood feeding behavior. Ramasamy et al. (2016) found that the evolution of olfactory genes is correlated with adaptation to new ecological niches by *Drosophila suzukii* and its close relative *Drosophila biarmipes*. They found that *D. suzukii* had a loss of function of ORs with affinities for volatiles produced during fermentation. They quantified the evolution of olfactory genes in *Drosophila* and revealed an array of genomic events that could be associated with the ecological adaptations of *D. suzukii*. Emeline et al. (2017) studied the differential expression of the OBP gene family in two closely related species of South American fruit flies, *Anastrepha fraterculus* and *A. obliqua*. They found eight OBP genes with differential expression between *A. fraterculus* and *A. obliqua*, suggesting that these genes have important roles in olfactory perception differences and accordingly are potentially related to species differentiation. Athrey et al. (2017) identified chemosensory gene families in olfactory organs of *Anopheles coluzzii* and *A. quadriannulatus* and inferred that divergence in OBP expression between the two species may be involved in differences in host preference.

Closely related species tend to feed on different host plants and to be polyphagous insects, with the exception of the closely related species *Eucryptorrhynchus scrobiculatus* and *E. brandti*. *E. scrobiculatus* and *E. brandti* feed on the same host but different parts of the host, and it is not clear whether chemosensory genes in the antennae affect their feeding differentiation. The role of olfaction in feeding differentiation is unknown. Yu et al. (2013) compared the antennal sensilla of both species to better understand their host-finding mechanism. In this study, we identified transcripts of OBPs, CSPs, ORs, IRs, GRs, and SNMPs in *E. scrobiculatus* and *E. brandti* antennae by high-throughput sequencing and investigated the expression patterns of OBP genes. Our results will be fundamental for studying the molecular mechanism of olfactory differentiation and provide a basis for understanding whether the differentiation of olfactory genes is related to feeding differences between the two species.

## Materials and Methods

### Ethics Statement

All of our experimental materials and methods are not contrary to ethics.

### Insect Rearing and Antennae Collection

Adults of *E. scrobiculatus* and *E. brandti* were collected in Xiaoxingdun village, Pingluo County, Ningxia Hui Autonomous Region (38°51′ 24″N, 106°31′ 38″E) in May 2017. *E. scrobiculatus* were reared in nylon mesh bags (80 × 40 cm) with 1-year-old branches and perennial branches of *A. altissima*. *E. brandti* were reared in mesh bags (80 × 40 cm) with the stems (*d* = 4 cm) of *A. altissima*. The mesh bags containing *E. scrobiculatus* and *E. brandti* were placed in separate breathable cartons, and the cartons were immediately taken to the laboratory in Beijing. In the laboratory, the adults were immediately frozen in liquid nitrogen and stored at -80°C until anatomical studies. The dissection was carried out on ice. The external genitalia were dissected to distinguish between males and females. The antennae were immediately cut from the bases of the heads of adults and added to a 2 mL centrifuge tube. RNA extraction was performed immediately after the dissection.

### Total RNA Extraction, cDNA Library Construction, and Illumina Sequencing

Fifteen and forty pairs of antennae of *E. scrobiculatus* and *E. brandti* were excised separately, and total RNA of female antennae (FA) and male antennae (MA) were extracted using the RNApure Total RNA Kit (Aidlab, Beijing, China). For each species and sex, data were obtained for three independent biological replicates, for a total of 12 samples. RNA was quantified using a NanoDrop 8000 (Thermo, Waltham, MA, United States). cDNA library construction and Illumina sequencing were performed at Bionova Biotechnology Co., Ltd. (Beijing, China). RNA quality was assessed by 1% agarose gel electrophoresis and analyzed using the 2100 Bioanalyzer (Agilent, Santa Clara, CA, United States). RNA was digested by DNase I to remove the DNA, and mRNA was enriched using oligo d(T). The mRNA was fragmented at a high temperature and reverse-transcribed. The resultant cDNA was subjected to purification, end repair, A-tailing, adapter ligation, and PCR amplification. The quality and quantity of the library were then evaluated using the Bioanalyzer 2100 and ABI StepOnePlus Real-Time PCR system (Applied Biosystems, Forester City, CA, United States), respectively. The qualified library was then used for high-throughput sequencing. These libraries were pair-end sequenced using the PE150 strategy on the Illumina HiSeq X Ten platform.

### Assembly, Functional Annotation, and Quantitative Expression Analysis

Raw reads were pre-processed by filtered adapters. Low-quality reads, including reads containing > 10% N (uncertain bases) and those with a median quality value (Q) ≤ 25, were removed to generate clean reads for subsequent analyses. Transcriptome assembly of each clean-read dataset for FA and MA was accomplished using Trinity (version: v2012-10-05) (Grabherr et al., 2011), with min_kmer_cov = 2. The Trinity outputs were clustered by TGICL (TIGR Gene Indices clustering tools) (Pertea et al., 2003). Consequently, six transcript levels were obtained, including those for FA and MA of *E. scrobiculatus* and *E. brandti* and the final transcript datasets for *E. scrobiculatus* and *E. brandti*.

Transcripts were annotated using the Trinotate pipeline^1^. All putative genes were searched using BLASTX and BLASTP against databases, including the Swissprot-Uniprot database, KOG (euKaryotic Ortholog Groups), GO (Gene Ontology), eggNOG (evolutionary genealogy of genes: Non-supervised Orthologous Groups), and KEGG (Kyoto Encyclopedia of Genes and Genomes) (*E*-value cut-off, 1e-5). According to the annotation results obtained using the KOG database, the unigenes were classified into 26 groups. Then, GO classification was performed using Blast2GO (Conesa et al., 2005).

Transcript abundances from RNA-seq data were quantified using RSEM (Li and Dewey, 2011). Transcript abundances were calculated as the FPKM (fragments per kilobase per million mapped fragments) (Trapnell et al., 2010).

### Identification of Chemosensory Genes

Candidate unigenes encoding OBPs, ORs, IRs, GRs, and SNMPs were found by keyword searches based on functional annotation results. Candidate unigenes encoding CSPs and pheromone-binding proteins (PBPs) were searched using BLASTX and tBLASTn according to downloaded sequences of *Dendroctonus ponderosae* CSPs (Andersson et al., 2013; Gu et al., 2015), *Colaphellus bowringi* CSPs (Li et al., 2015), and coleopteran PBPs against the local transcriptomes. All putative unigenes were confirmed by BLASTX searches against the NCBI non-redundant protein sequences database. Open reading frames (ORFs) of candidate genes were identified using ORF Finder and verified using tBLASTn in NCBI. The putative N-terminal signal peptides of candidate OBPs and CSPs were predicted using SignalP 4.1 server version with default parameters (Nielsen, 2017). The transmembrane domains of candidate ORs, IRs, GRs, and SNMPs were predicted using TMHMM server version 2.0 (Krogh et al., 2001).

### Sequence and Phylogenetic Analysis

Amino acid sequences of candidate OBPs, CSPs, ORs, IRs, GRs, and SNMPs were aligned using ClustalX and further edited using GeneDoc. The phylogenetic trees of *E. scrobiculatus* and *E. brandti* chemosensory genes were constructed using the neighbor-joining method in MEGA 6.0 with default settings and 1000 bootstrap replicates. The dendrograms were color-coded and arranged using FigTree v1.4.3. The sequence identities of these chemosensory genes between *E. scrobiculatus* and *E. brandti* were determined using ClustalX and BLASTP.

### Tissue- and Sex-Specific Expression of Candidate OBP Genes

The expression patterns of OBP genes in both female and male tissues (antennae, rostrum, leg, and head without the antennae and rostrum) of *E. scrobiculatus* and *E. brandti* were analyzed by RT-qPCR using a Bio-Rad CFX Connect PCR system (Bio-Rad, Hercules, CA, USA). Female and male antennae, rostrum, legs, and heads (without the antennae and rostrum) (female:male = 1:1) were collected from adult *E. scrobiculatus* and *E. brandti*. Total RNA was extracted using the RNApure Total RNA Kit (Aidlab, Beijing, China). The cDNA was synthesized from total RNA using the TRUEscript 1st Strand cDNA Synthesis Kit (Aidlab). Gene-specific primers were designed using Primer3Plus v2.4.2 (Supplementary Table S9). Each reaction was run in triplicate with three biological duplications, and PCRs with no template were used as controls. *α-Tubulin* and ribosomal protein (*RPS11*) were used as reference genes for *E. scrobiculatus* and *E. brandti*, respectively. Each RT-qPCR contained 10 μL of TB Green Premix Ex Taq II (Takara, Beijing, China), 1 μL of each primer, 2 μL of sample cDNA, and 6 μL of sterilized H_2_O. RT-qPCR cycling parameters were 95°C for 2 min, followed by 40 cycles of 95°C for 5 s and 60°C for 30 s. The melting curve was analyzed to evaluate the specificity of primers after each reaction, and the 2^-ΔΔct^ method was used to calculate the relative expression levels of OBP genes. The specificity of primers for each target gene was validated. RT-qPCR data were analyzed and plotted using GraphPad Prism 5.0 (GraphPad Software, La Jolla, CA, United States). The differences in the relative expression levels of OBPs were calculated using SPSS19.0 (SPSS Inc., Chicago, IL, United States) by a one-way nested analysis of variance (ANOVA), followed by Duncan’s new multiple range test (α = 0.05).

## Results

### Antennal Transcriptome Sequencing and Sequence Assembly

We sequenced the transcriptomes of FA and MA of *E. scrobiculatus* and *E. brandti* with three independent biological replicates. We obtained approximately 48.78 (FA-1), 48.44 (FA-2), 48.44 (FA-3), 49.86 (MA-1), 48.63 (MA-2), and 49.50 (MA-3) million clean reads from *E. scrobiculatus*. These were assembled into 31757 unigenes for females and 32923 for males. A final transcript dataset (ES) with 46380 unigenes was obtained by TGICL, with a mean length of 3134 bp and N50 of 4850 bp (Supplementary Table S1). Similarly, we obtained approximately 41.02 (FA-1), 49.09 (FA-2), 49.33 (FA-3), 48.47 (MA-1), 49.38 (MA-2), and 49.22 (MA-3) million clean reads from *E. brandti*. These were assembled into 44720 unigenes for females and 37712 for males. A final transcript dataset (EB) with 56084 unigenes was obtained by TGICL, with a mean length of 2130 bp and N50 of 3396 bp (Supplementary Table S1). The datasets of transcriptomes during the current study have been uploaded to the NCBI SRA database (accession number: SRP155112).

### Functional Annotation

To acquire more comprehensive sequence information, all six transcript sets were annotated. In total, 29631 (63.9%) and 26487 (47.2%) unigenes from *E. scrobiculatus* and *E. brandti* were annotated by BLASTX, respectively. For both *E. scrobiculatus* and *E. brandti*, the number of annotated unigenes in the final transcript dataset (ES and EB) was significantly higher than that of female antennal transcripts (ESF and EBF) and male antennal transcripts (ESM and EBM) (Supplementary Table S2).

We classified 28725 (61.9%) unigenes in *E. scrobiculatus* and 22839 (40.7%) in *E. brandti* into 26 KOG protein groups. The four largest groups were general function prediction only, signal transduction mechanisms, function unknown, and post-translational modification/protein turnover/chaperones. Very few unigenes were assigned to the nuclear structure and unnamed protein group (Supplementary Figure S1).

Gene ontology annotation was used to classify unigenes into different functional categories. Of the 46380 unigenes in *E. scrobiculatus*, 30714 (66.2%) could be annotated based on sequence similarity. We assigned 27666 of the 56084 *E. brandti* antennal unigenes (49.3%) to specific GO terms. The distributions of GO terms in the three major categories were similar in the two species. In the biological processes category, “cellular process” and “metabolic process” were most highly represented. In the cellular components category, the most abundant GO terms were “cell,” “cell part,” and “organelle.” In the molecular function category, “binding” and “catalytic activity” were the most highly represented (Supplementary Figure S2).

### Candidate OBPs in *E. scrobiculatus* and *E. brandti*

Based on our analysis of the antennal transcriptomes in the two species, we identified 31 and 28 candidate OBP genes in the female antennal, male antennal, and combined female and male datasets for *E. scrobiculatus* and *E. brandti*, respectively. All but one transcript (EscrOBP31) had complete ORFs, and 27 transcripts of EscrOBPs and 23 transcripts of EbraOBPs included predicted signal peptide sequences. Detailed information was reported in Supplementary Table S3.

**FIGURE 1:**
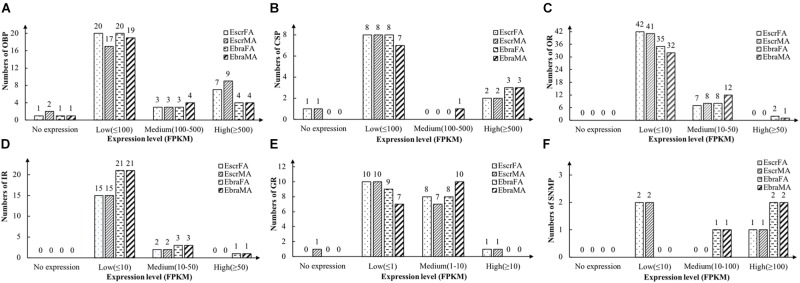
Number of chemosensory genes at different expression levels in female and male antennae of *E. scrobiculatus* and *E. brandti*. Expression level is expressed as fragments per kilobase per million mapped fragments (FPKM). **(A)** OBP; **(B)** CSP; **(C)** OR; **(D)** IR; **(E)** GR; **(F)** SNMP.

Odorant-binding proteins with FPKM values of ≥ 500 were defined as highly expressed genes, those with values 100–500 were defined as moderately expressed genes, and those with values of ≤ 100 were defined as weakly expressed genes. Most OBPs were weakly expressed, but a few OBPs were highly expressed in antennae, and more genes were highly expressed in *E. scrobiculatus* than in *E. brandti* (Figure 1A and Supplementary Table S3).

**FIGURE 2:**
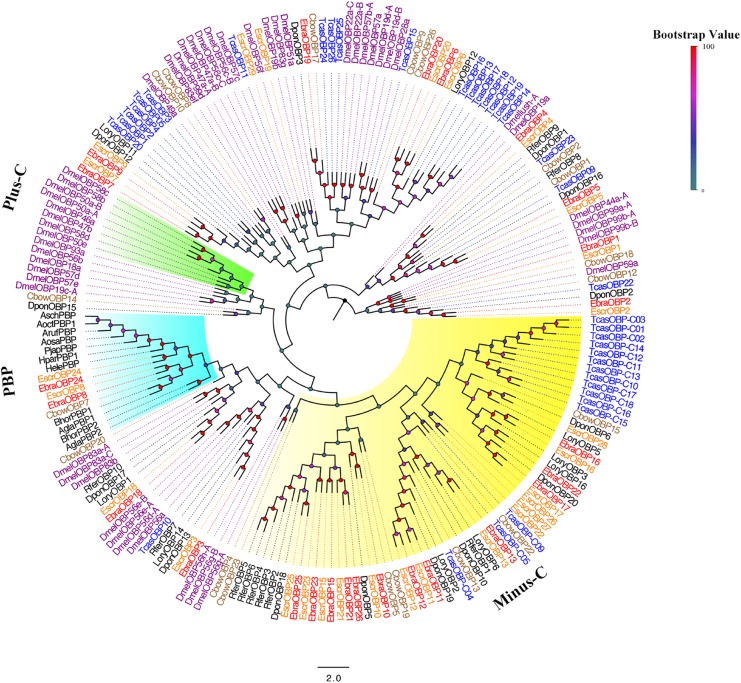
Phylogenetic tree of putative odorant binding protein (OBP) genes. The *E. scrobiculatus* genes are shown in yellow and *E. brandti* genes are shown in red. The tree was constructed using MEGA6 with Neighbor-joining method. Amino acid sequences used for the tree are listed in Supplementary Table S10.

A phylogenetic tree was built using the newly obtained sequences and those from Diptera and Coleoptera. Among EscrOBPs, 13 showed the classic motif of six conserved cysteines, 15 were Minus-C, and 3 were undefined owing to less conserved cysteines. For EbraOBPs, we found 13 classic and 13 Minus-C OBPs; two were undefined (Figure 2 and Supplementary Figure S3). Remarkably, *EscrOBP8*, *EscrOBP24* and *EbraOBP8*, *EbraOBP24* formed a cluster with other coleopteran PBPs, with sequence similarities of 99 and 86%, respectively. We inferred that *EscrOBP8* (*EscrPBP1*), *EscrOBP24* (*EscrPBP2*) and *EbraOBP8* (*EbraPBP1*), *EbraOBP24* (*EbraPBP2*) were putative PBPs from *E. scrobiculatus* and *E. brandti*, respectively. Most OBPs clustered with other coleopteran OBPs, except for *EscrOBP19* and *EscrOBP29*. In the phylogenetic tree, we detected 20 OBP pairs in *E. scrobiculatus* and *E. brandti* with high homology (Figure 2). We evaluated the sequence identity between EscrOBPs and EbraOBPs by ClustalX and BLASTP and found that 18 OBP orthologous pairs shared amino acid identities of ≥ 90% between *E. scrobiculatus* and *E. brandti* (Supplementary Table S8).

### Candidate CSPs in *E. scrobiculatus* and *E. brandti*

We identified 11 different transcripts encoding candidate CSPs in *E. scrobiculatus* and *E. brandti*. All but one transcript (EbraCSP7) included full-length ORFs, and 9 EscrCSPs and 10 EbraCSPs had predicted signal peptide sequences (Supplementary Table S4). All of the identified amino acid sequences possessed the highly conserved four-cysteine profile (Supplementary Figure S4). Most CSPs exhibited low expression levels; two EscrCSPs and three EbraCSPs were highly expressed in antennae (Figure 1B and Supplementary Table S4).

A phylogenetic tree was built using all of these CSPs and those of Lepidoptera and Coleoptera. EscrCSPs and EbraCSPs clustered with other coleopteran CSPs, and no specific CSP lineages were evident (Supplementary Figure S5). We detected 10 CSP orthologous pairs sharing amino acid similarities of ≥ 90% between *E. scrobiculatus* and *E. brandti* (Supplementary Table S8).

### Candidate ORs in *E. scrobiculatus* and *E. brandti*

We identified 49 transcripts for putative ORs in *E. scrobiculatus* and 45 in *E. brandti*. Of these, 42 EscrORs and 36 EbraORs contained complete ORFs encoding proteins of more than 300 amino acids, with 1–8 transmembrane domains. Furthermore, 16 EscrORs and 10 EbraORs encoded seven-transmembrane-domain proteins. In comparisons with OR genes from other insect species by BLASTX, we found that all putative EscrORs shared identities of 26 and 92% with other ORs, with almost identical values (27–92%) for EbraORs. Detailed information is reported in Supplementary Table S5. Expression levels of ORs in *E. scrobiculatus* and *E. brandti* were similar; most EscrORs and EbraORs were weakly expressed (FPKM ≤ 10) in antennae. Note that *EbraOR24* (*EbraOrco*) was highly expressed while *EscrOR24* (*EscrOrco*) was weakly expressed (Figure 1C and Supplementary Table S5).

**FIGURE 3:**
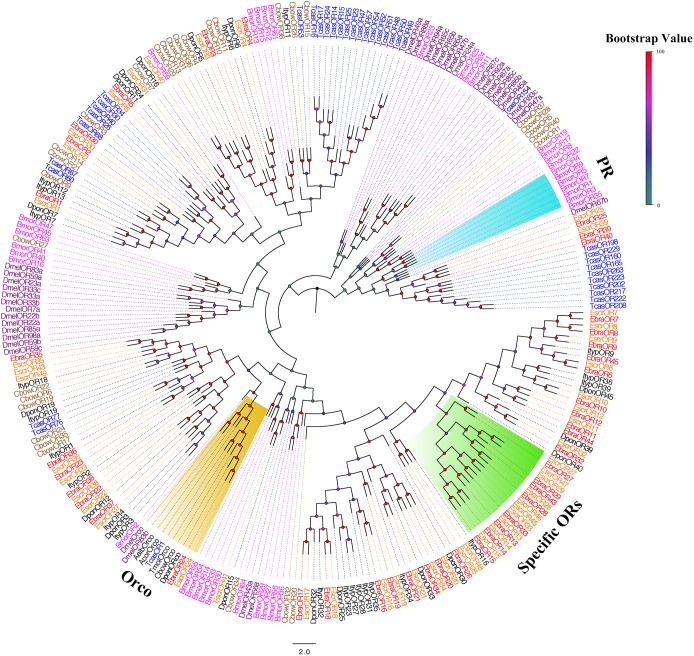
Phylogenetic tree of putative odorant receptor (OR) genes. The *E. scrobiculatus* genes are shown in yellow and *E. brandti* genes are shown in red. The tree was constructed using MEGA6 with Neighbor-joining method. Amino acid sequences used for the tree are listed in Supplementary Table S10.

We performed a phylogenetic analysis using our candidate ORs and those from Lepidoptera, Diptera, and Coleoptera. *EscrOR24* and *EbraOR24* clustered with *DmelOR83b*, the highly conserved co-receptor Orco (Larsson et al., 2004; Hallem et al., 2006). Sequence identity between *EscrOR24* and *EbraOR24* was very high (98%). No candidate ORs clustered with *DmelOR67d*, the pheromone receptors (PRs) from *D. melanogaster*, and other PRs. Within these OR sequences, we found a species-specific clade including 10 EscrORs (*EscrOR13*, *14*, *15*, *27*, *28*, *29*, *33*, *45*, *46*, and *47*) and 8 EbraORs (*EbraOR13*, *14*, *15*, *27*, *28*, *29*, *33*, and *43*) sharing low homology with other coleopteran ORs (Figure 3). These genes may be related to the detection of the characteristic volatile of *A. altissima*.

We compared the amino acid sequences of EscrORs and EbraORs. Sequence similarities of the 39 pairs of homologous ORs were greater than 70%. In addition, 25 OR orthologous pairs shared amino acid identities of ≥ 90% between *E. scrobiculatus* and *E. brandti*. The higher sequence similarity in homologous ORs indicated they may be involved in olfactory recognition of *A. altissima* (Supplementary Table S8).

### Candidate IRs in *E. scrobiculatus* and *E. brandti*

We identified 17 candidate IRs in *E. scrobiculatus* and 25 in *E. brandti*. All but three transcripts (*EscrIR2*, *9* and *EbraIR2*) contained full-length ORFs with one to five transmembrane domains (Supplementary Table S6). Expression levels of IRs were the same as the levels of ORs in *E. scrobiculatus* and *E. brandti*. Two EscrIRs and three EbraIRs were moderately expressed, *EbraIR6* was highly expressed (Figure 1D and Supplementary Table S6).

**FIGURE 4:**
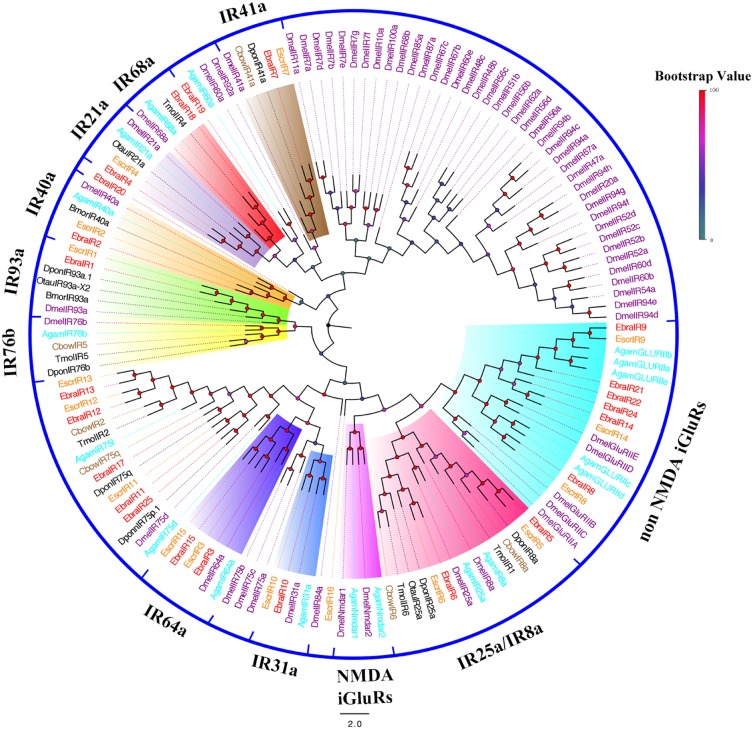
Phylogenetic tree of putative ionotropic receptor (IR) genes. The *E. scrobiculatus* genes are shown in yellow and *E. brandti* genes are shown in red. The tree was constructed using MEGA6 with Neighbor-joining method. Amino acid sequences used for the tree are listed in Supplementary Table S10.

To further infer the function of IR genes, a phylogenetic tree was constructed using these sequences and homologous sequences in Lepidoptera, Diptera, and Coleoptera. For EscrIRs, seven EscrIRs (*EscrIR1*, *2*, *3*, *15*, *4*, *7*, and *10*) clustered with the presumed “antennal” orthologues *IR93a*, *40a*, *64a*, *21a*, *41a*, and *31a*. *EscrIR5* and *EscrIR6* were distributed in the *IR8a* and *IR25a* groups, which are co-receptors (Abuin et al., 2011). *EscrIR8*, *9*, and *14* clustered with the non-NMDA iGluRs group (Croset et al., 2010). For EbraIRs, eight EbraIRs (*EbraIR1*, *2*, *3*, *15*, *4*, *7*, *10*, *18*) clustered with the presumed “antennal” orthologues *IR93a*, *40a*, *64a*, *21a*, *41a*, *31a*, and *68a*. *EbraIR8*, *9*, *14*, *21*, *22*, and *24* formed a group with non-NMDA iGluRs. Notably, the conserved “antennal” orthologues *IR60a* and *IR76b* were lacking in the *E. scrobiculatus* and *E. brandti* transcriptomes, while *IR68a* was only absent from *E. scrobiculatus*. Notably, *EscrIR16*, *EbraIR19*, and *EbraIR20* were divergent compared with other DmelIRs sharing low homology (Figure 4). Fifteen IR orthologous pairs shared amino acid similarities of > 85% between *E. scrobiculatus* and *E. brandti*. The similarity of nine pairs of IRs exceeded 95%. These IRs may play a key role in host recognition (Supplementary Table S8).

### Candidate GRs in *E. scrobiculatus* and *E. brandti*

We identified 19 candidate GRs from transcript datasets of *E. scrobiculatus*, and 18 transcripts contained complete ORFs. Similarly, we identified 17 candidate GRs in *E. brandti*, and 11 transcripts contained complete ORFs (Supplementary Table S7). Expression levels of GRs were generally lower than those of other chemosensory genes. GRs with FPKM values ≤ 1 were defined as having low expression, those with values of 1–10 were defined as moderately expressed, and those with values ≥ 10 were defined as highly expressed. The majority of GR genes were weakly and moderately expressed in *E. scrobiculatus* and *E. brandti*; only one EscrGR (*EscrGR4*) was highly expressed (Figure 1E and Supplementary Table S7).

**FIGURE 5:**
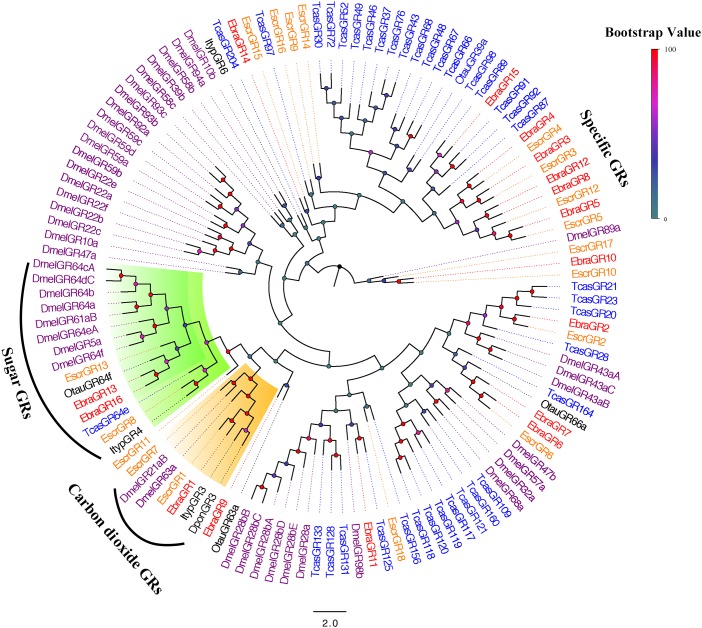
Phylogenetic tree of putative gustatory receptor (GR) genes. The *E. scrobiculatus* genes are shown in yellow and *E. brandti* genes are shown in red. The tree was constructed using MEGA6 with Neighbor-joining method. Amino acid sequences used for the tree are listed in Supplementary Table S10.

All of these protein sequences and the sequences from five additional insect species were used to construct a phylogenetic tree. We found that *EscrGR8*, *Escr13*, *EbraGR13*, and *EbraGR16* were members of the sugar-receptor subfamily, and *EscrGR1*, *EscrGR7*, and *EbraGR1* were assigned to the CO_2_-receptor subfamily, indicating that these GRs might be related to the detection of CO_2_ (Jones et al., 2007; Kwon et al., 2007) and sugar (Dahanukar et al., 2007; Jiao et al., 2008; Sato et al., 2011). Furthermore, we found a species-specific clade including four GRs from *E. scrobiculatus* (*EscrGR3*, *4*, *5*, and *12*) and five from *E. brandti* (*EbraGR3*, *4*, *5*, *8*, and *12*) that shared low homology with other coleopteran GRs (Figure 5). The amino acid sequences of *E. scrobiculatus* and *E. brandti* for five pairs of homologous GRs had similarities of more than 90% (Supplementary Table S8).

### Candidate SNMPs in *E. scrobiculatus* and *E. brandti*

We identified three transcripts encoding candidate SNMPs in *E. scrobiculatus* and *E. brandti* (Supplementary Table S7). Two EscrSNMPs were weakly expressed (FPKM ≤ 10), one EbraSNMP was moderately expressed (FPKM, 10–100), and one EscrSNMP and two EbraSNMPs were highly expressed (FPKM ≥ 100) (Figure 1F and Supplementary Table S7).

Based on a phylogenetic analysis, *EscrSNMP1*, *EscrSNMP3*, *EbraSNMP1*, and *EbraSNMP3* were very similar to *DmelSNMP1*, which encodes a protein required for correct pheromone detection (Rogers et al., 2001; Benton et al., 2007; Jin et al., 2008). *EscrSNMP2* and *EbraSNMP2* were similar to *DmelSNMP2*, which is expressed in supporting cells (Nichols and Vogt, 2008; Forstner et al., 2008; Gu et al., 2013) (Supplementary Figure S6). Three SNMP orthologous pairs shared amino acid identities of > 90% between *E. scrobiculatus* and *E. brandti* (Supplementary Table S8).

### Tissue- and Sex-Specific Expression of Candidate *E. scrobiculatus* and *E. brandti* OBP Genes

**FIGURE 6:**
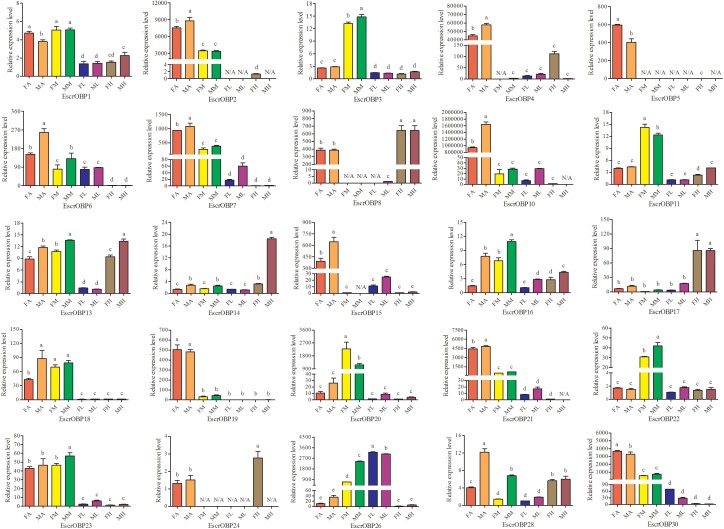
Relative expression levels of *E. scrobiculatus* OBPs in different tissues of female and male adults by RT-qPCR. FA, female antennae; MA, male antennae; FM, female rostrums; MM, male rostrums; FL, female legs; ML, male legs; FH, female head (with antennae and rostrum cut off); MH, male head (with antennae and rostrum cut off). The bar represents standard error and the different small letters (a–e) above each bar indicate significant differences (*P* < 0.05). N/A indicates that the transcript level is too low to measure.

**FIGURE 7:**
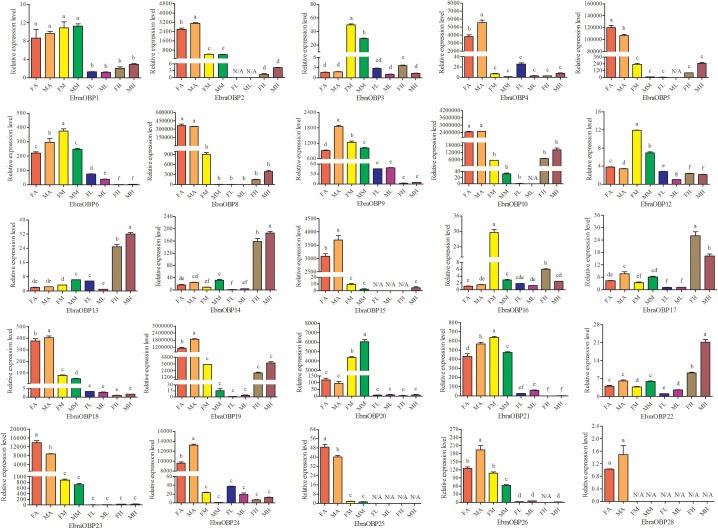
Relative expression levels of *E. brandti* OBPs in different tissues of female and male adults by RT-qPCR. FA, female antennae; MA, male antennae; FM, female rostrums; MM, male rostrums; FL, female legs; ML, male legs; FH, female head (with antennae and rostrum cut off); MH, male head (with antennae and rostrum cut off). The bar represents standard error and the different small letters (a–g) above each bar indicate significant differences (*P* < 0.05). N/A indicates that the transcript level is too low to measure.

Expression patterns of 25 OBPs in female and male antennae, rostrum, legs, and heads (excluding the antennae and rostrum) from *E. scrobiculatus* and *E. brandti* were determined by RT-qPCR. All OBPs were expressed in antennae in both species. In *E. scrobiculatus*, we detected high expression of 11 putative OBP genes (*EscrOBP2*, *4*, *5*, *6*, *7*, *10*, *15*, *19*, *21*, *28*, and *30*) in the antennae. *EscrOBP5* and *EscrOBP30* were significantly female-biased, and the antennal expression of eight OBPs (*EscrOBP2*, *4*, *6*, *7*, *10*, *15*, *21*, and *28*) was significantly male-biased. Furthermore, we detected significantly higher expression of four OBPs (*EscrOBP3*, *11*, *20*, and *22*) in the rostrum than in other tissues, and we detected significantly greater expression of four OBPs (*EscrOBP8*, *14*, *17*, and *24*) in the head. Interestingly, *EscrPBP1* (*EscrOBP8*) and *EscrPBP2* (*EscrOBP24*) were more highly expressed in the head than in the antennae. In addition, we observed higher levels of four OBPs (*EscrOBP1*, *16*, *18*, and *23*) in the antennae and rostrum than in other tissues, *EscrOBP13* was highly expressed in all tissues except for the leg, and *EscrOBP26* showed higher expression in the leg than in other tissues (Figure 6). In *E. brandti*, we detected significantly higher expression of 14 putative OBPs (*EbraOBP2*, *4*, *5*, *8*, *9*, *10*, *15*, *18*, *19*, *23*, *24*, *25*, *26*, and *28*) in the antennae than in other tissues. Antennal expression levels of *EbraOBP5*, *EbraOBP23* and *EbraOBP25* were female-biased, and the expression of nine OBPs (*EbraOBP2*, *4*, *9*, *15*, *18*, *19*, *24*, *26*, and *28*) was male-biased. We observed significantly higher levels of five OBPs (*EbraOBP3*, *6*, *12*, *16*, and *21*) in the rostrum than in other tissues and significantly higher expression of four OBPs (*EbraOBP13*, *14*, *17*, and *22*) in the head. Four OBPs (*EbraOBP1*, *9*, *21*, and *26*) showed higher expression in the antennae and rostrum than in other tissues (Figure 7).

## Discussion

The weevils *E. scrobiculatus* and *E. brandti* are sympatric and closely related, feeding on the same host (*A. altissima*) but different parts. It is not clear whether olfaction plays an important role in feeding differentiation. We analyzed the antennal transcriptomes of *E. scrobiculatus* and *E. brandti* and searched for chemosensory genes to evaluate interspecific differences in olfactory genes.

In this study, we sequenced female and male antennal transcriptomes of *E. scrobiculatus* and *E. brandti* using the Illumina HiSeq X Ten platform, assembled reads using Trinity, and performed a clustering analysis using TGICL. We acquired and annotated female and male antennal transcripts and final transcripts of *E. scrobiculatus* and *E. brandti*. We detected more unigenes in *E. brandti* (56084) than in *E. scrobiculatus* (46380), but the mean length and N50 of unigenes in *E. brandti* were lower than those in *E. scrobiculatus*. Additionally, the number of annotated unigenes in *E. brandti* (47.2%) was much less than that in *E. scrobiculatus* (63.9%). These results suggest that the *E. brandti* genome contains more species-specific genes than the *E. scrobiculatus* genome. We searched six annotated databases and identified 130 putative chemosensory genes (31 OBPs, 11 CSPs, 49 ORs, 17 IRs, 19 GRs, and 3 SNMPs) in *E. scrobiculatus* and 129 (28 OBPs, 11 CSPs, 45 ORs, 25 IRs, 17 GRs, and 3 SNMPs) in *E. brandti*, fewer than the number of chemosensory genes identified in *Rhynchophorus ferrugineus* (Antony et al., 2016) and more than those in *Dendroctonus valens* (Gu et al., 2015), *Ips typographus* and *Dendroctonus ponderosae* (Andersson et al., 2013), and *Tomicus yunnanensis* (Liu et al., 2018) (Table 1).

**Table 1:** The number of odorant binding protein (OBP), chemosensory protein (CSP), odorant receptor (OR), ionotropic receptor (IR), gustatory receptor (GR), and sensory neuron membrane protein (SNMP) in Curculionoidea.

	OBP	CSP	OR	IR	GR	SNMP	Total
*D. valens*	21	6	22	3	4	4	60
*Dendroctonus armandi*	11 (unpublished)	9	–	–	–	–	20
*Ips acuminatus*	1 (unpublished)	–	–	–	–	–	1
*I. typographus*	15	6	43	7	6	3	80
*D. ponderosae*	31	11	49	15	2	3	111
*Tomicus yunnanensis*	45	12	9	3	8	3	80
*Lissorhoptrus oryzophilus*	10	5	–	–	–	–	15
*Callosobruchus chinensis*	21	5	–	–	–	–	26
*R. ferrugineus*	38	12	77	10	15	6	158
*Anthonomus grandis*	–	12 (unpublished)	–	–	3 (unpublished)	5 (unpublished)	20
*E. scrobiculatus*	31	11	49	17	19	3	130
*E. brandti*	28	11	45	25	17	3	129


We detected more candidate OBPs in the two weevils (31 in *E. scrobiculatus* and 28 in *E. brandti*) than previously reported in *D. valens* (21 OBPs) (Gu et al., 2015), *I. typographus* (15 OBPs) (Andersson et al., 2013), and *L. oryzophilus* (10 OBPs) (Yuan et al., 2016), but fewer OBPs than in *R. ferrugineus* (38 OBPs) (Antony et al., 2016) and *Tribolium castaneum* (49 OBPs) (Dippel et al., 2014). Weevils and *D. ponderosae* had similar numbers of OBPs (31 OBPs) (Andersson et al., 2013). Similar results were obtained for CSPs (Table 1). Chemosensory receptors play a critical role in the reception of chemicals from the environment and the regulation of insect behaviors. However, there are few known receptors in Curculionoidea, including ORs, IRs, and GRs, especially IRs and GRs. In this study, we identified 49 transcripts for putative ORs in *E. scrobiculatus* and 45 in *E. brandti*, compared with 22 ORs in *D. valens* (Gu et al., 2015), 43 in *I. typographus* (43 ORs), and 77 in *R. ferrugineus* (Antony et al., 2016). There were substantially more IRs and GRs in weevils than in *D. valens* (3 IRs and 4 GRs) (Gu et al., 2015), *R. ferrugineus* (10 IRs and 15 GRs) (Antony et al., 2016), *I. typographus* (7 IRs and 6 GRs), and *D. ponderosae* (15 IRs and 2 GRs) (Andersson et al., 2013). These differences could be due to differences in sample preparation and sequencing methods or the evolution of divergent physiological behaviors in distinct environments (Goldman-Huertas et al., 2015).

Despite increasing research on insect olfaction mechanisms, little is known about chemoreception in coleopterans, especially Curculionidae, compared with Lepidoptera and Diptera. Owing to the limited functional information for coleopterans, we inferred the physiological functions of chemosensory genes of *E. scrobiculatus* and *E. brandti* using a phylogenetic approach. Most OBPs in *E. scrobiculatus* and *E. brandti* clustered with those of other coleopterans with high homology, except for *EscrOBP19* and *EscrOBP29*, which need to be further studied. All candidate EscrCSPs and EbraCSPs shared high sequence identity with CSPs of other coleopteran insects, and no species-specific CSP was found. We identified species-specific OR transcripts in *E. scrobiculatus* and *E. brandti*, and these may play important roles in recognizing specific volatiles of *A. altissima*. We found “antennal IR” orthologues in *E. scrobiculatus* and *E. brandti*, such as *IR21a*, *IR31a*, *IR40a*, *IR41a*, *IR93a*, *IR64a*, and co-receptor *IR8a/IR25a*. Moreover, we detected “divergent IR” orthologues in *E. brandti*, *EbraIR19*, and *EbraIR20*, which may act as GRs in distinct taste organs and stages of *E. brandti* (Croset et al., 2010). We detected more non-NMDA iGluRs in *E. brandti* than in *E. scrobiculatus*. Neither species had NMDA iGluRs, which are related to fast excitatory synaptic transmission in vertebrates and invertebrates (Littleton and Ganetzky, 2000; Tikhonov and Magazanik, 2009). The effects of non-NMDA iGluRs on *E. scrobiculatus* and *E. brandti* need to be further studied. Despite similar numbers of candidate GRs in *E. scrobiculatus* and *E. brandti*, their amino acid identities were lower than those for other chemosensory genes, and this could explain their different feeding habits. In the future, we intend to explore the expression pattern and function of these GRs in the two weevils, which will be helpful to study their feeding differentiation.

Based on the expression levels of chemosensory genes in *E. scrobiculatus* and *E. brandti* and their phylogenetic analysis, we found some differential expressed genes. Orco is highly conserved in insects, while the expression level of putative *Orco* in *E. scrobiculatus* and *E. brandti* (*EscrOR24* and *EbraOR24*) was quite different. *EbraOR24* (*EbraOrco*) was highly expressed while *EscrOR24* (*EscrOrco*) was weakly expressed in antennae. The number of putative IRs in *E. brandti* was more than that in *E. scrobiculatus*, so is the highly-expressed genes. *EscrIR1* and *EscrIR6* were moderately expressed, while *EbraIR5*, *EbraIR17*, and *EbraIR25* were moderately expressed, and *EbraIR6* was highly expressed in antennae. *EscrIR6*, *EbraIR5*, and *EbraIR6* clustered with the *IR8a*/*IR25a* group. *EscrGR4* and *EbraGR4* were species-specific GRs in phylogenetic analysis, *EscrGR4* was highly expressed in antennae, while EbraGR4 was weakly expressed. These differential expressed genes may play the role in olfactory differentiation of two weevils, their functions need to be further studied in the future.

We investigated the expression profile of OBPs in the two weevils by RT-qPCR. Some OBPs with high amino acid sequence similarities exhibited similar expression in various tissues of *E. scrobiculatus* and *E. brandti*, including *EscrOBP1*, *EscrOBP2*, and *EscrOBP3* and *EbraOBP1*, *EbraOBP2*, and *EbraOBP3*, indicating that these OBPs may be involved in the detection of the same host odors. Some OBPs with high amino acid sequence similarity exhibited expression differences in various tissues between *E. scrobiculatus* and *E. brandti*. For example, *EscrOBP13* was highly expressed in the antennae, rostrum, and head, while *EbraOBP13* was highly expressed in the head. Notably, *EscrOBP8* and *EscrOBP24* were highly expressed in the head and *EscrOBP24* was female-biased, while *EbraOBP8* and *EbraOBP24* were highly expressed in the antennae and *EbraOBP24* was male-biased, suggesting that these genes could have different binding affinities for pheromone compounds (Plettner et al., 2000).

Growing evidence indicates that chemosensory genes play key roles in host specialization in insects (Visser, 1986; Whiteman and Pierce, 2008; Schymura et al., 2010; Eyres et al., 2017). Of these genes, OBPs and CSPs are small, highly conserved families, mainly involved in ligand binding to receptors. By contrast, ORs and GRs are large and rapidly evolving gene families. Many studies have emphasized the roles of chemoreceptors in differences between host-associated species (Hallem et al., 2006; McBride, 2007; Smadja et al., 2012; McBride et al., 2014). For example, Eyres et al. (2017) confirmed that differences in chemosensory genes were important for the divergence of pea aphid races, especially GRs and ORs. Interestingly, most candidate OBPs and CSPs in *E. scrobiculatus* and *E. brandti* clustered with other coleopteran genes, and no species-specific clade was found, indicating that these genes were conserved. We identified species-specific clades of ORs and GRs in *E. scrobiculatus* and *E. brandti*, which might correspond to the odor of the specific host, providing evidence for olfactory differentiation in weevils. In future research, we plan to further study the function of ORs and GRs in *E. scrobiculatus* and *E. brandti* to explore the reason for their feeding differentiation.

In this study, we identified and compared putative chemosensory genes in antennae of *E. scrobiculatus* and *E. brandti*. Sequence identity between *E. scrobiculatus* and *E. brandti* was > 90% for more than half of the genes. These genes were likely to be related to the specific feeding on *A. altissima*. We also found species-specific genes in *E. scrobiculatus* and *E. brandti*; these genes might play a critical role in olfactory and feeding differentiation. These data provide a foundation for studying the molecular mechanism of olfaction and olfactory differentiation in weevils as well as a theoretical basis for the differentiation of the closely related species.

## Author Contributions

XW and JW conceived and designed the experiments. XW, QW, and PG performed the experiments. XW and QW analyzed the data. XW wrote the manuscript. All authors reviewed the final manuscript and approved the submitted version.

## Conflict of Interest Statement

The authors declare that the research was conducted in the absence of any commercial or financial relationships that could be construed as a potential conflict of interest.

## Footnotes

1 https://trinotate.github.io/
