# Supplementary material for: Identification and Comparison of Chemosensory Genes in the Antennal Transcriptomes of Eucryptorrhynchus scrobiculatus and E. brandti Fed on Ailanthus altissima
Source: Front Physiol. 2018 Nov 20;9:1652. doi: 10.3389/fphys.2018.01652 (PMC6256065; doi:10.3389/fphys.2018.01652)
Supplement: Supplementary file 1 [file Data_Sheet_1.PDF]

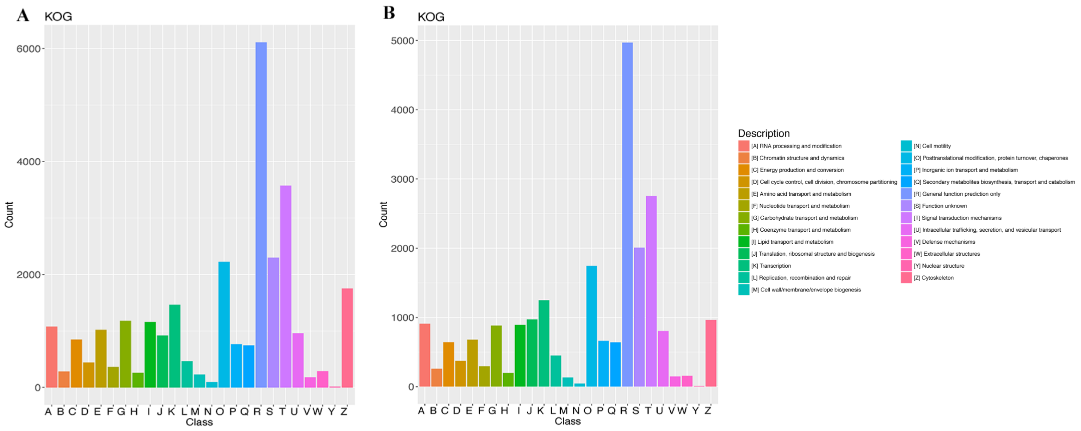

Figure S1 euKaryotic Ortholog Groups (KOG) classification of unigenes. (A) *E. scrobiculatus*. (B) *E. brandti*.

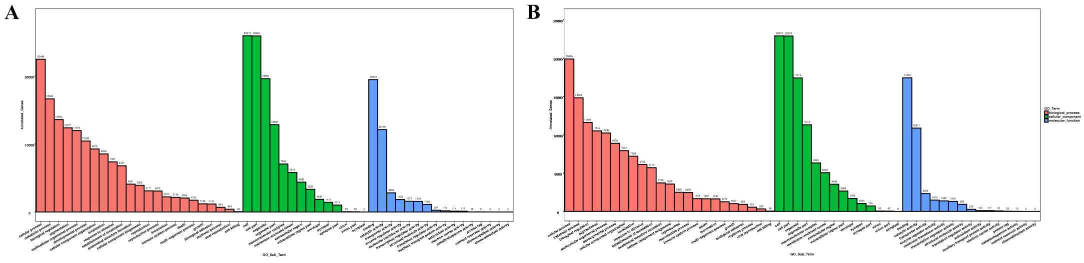

Figure S2 Gene ontology (GO) classification of unigenes. (A) *E. scrobiculatus*. (B) *E. brandti*.

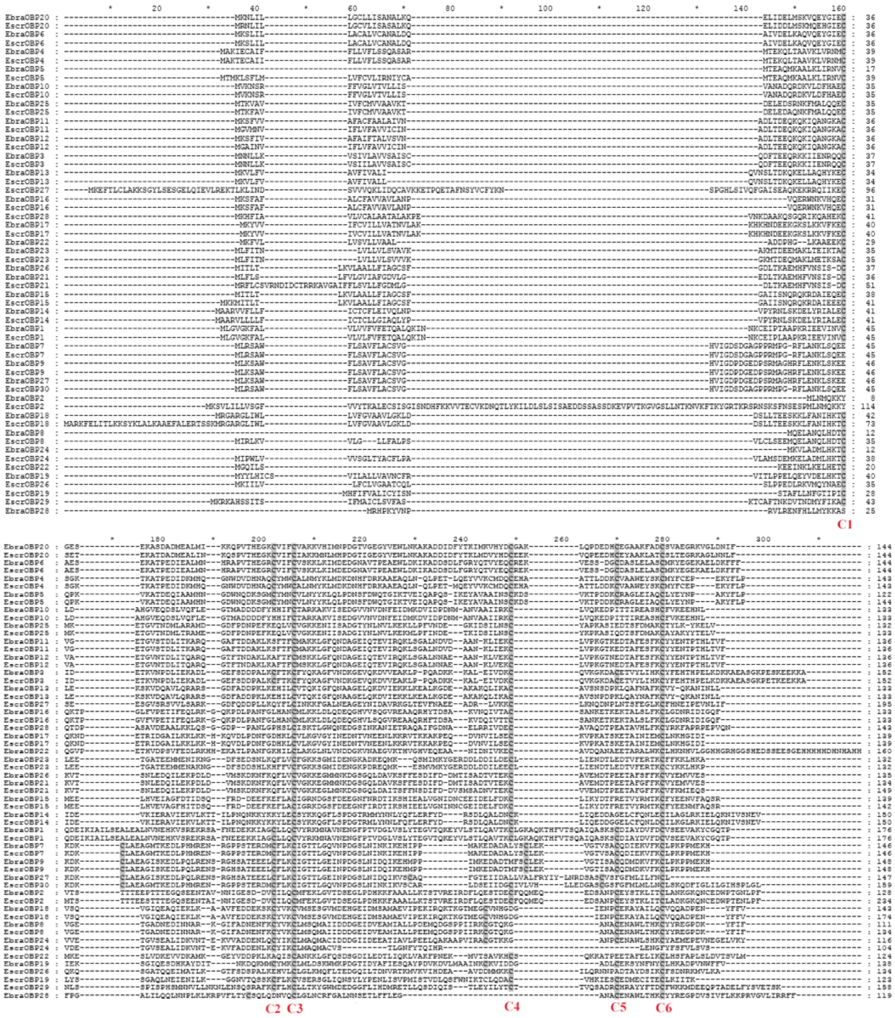

Figure S3 Alignment of amino acid sequences of odorant binding proteins (OBPs) of *E. scrobiculatus* and *E. brandti*. Conserved cysteine sites are marked with red capital letter.

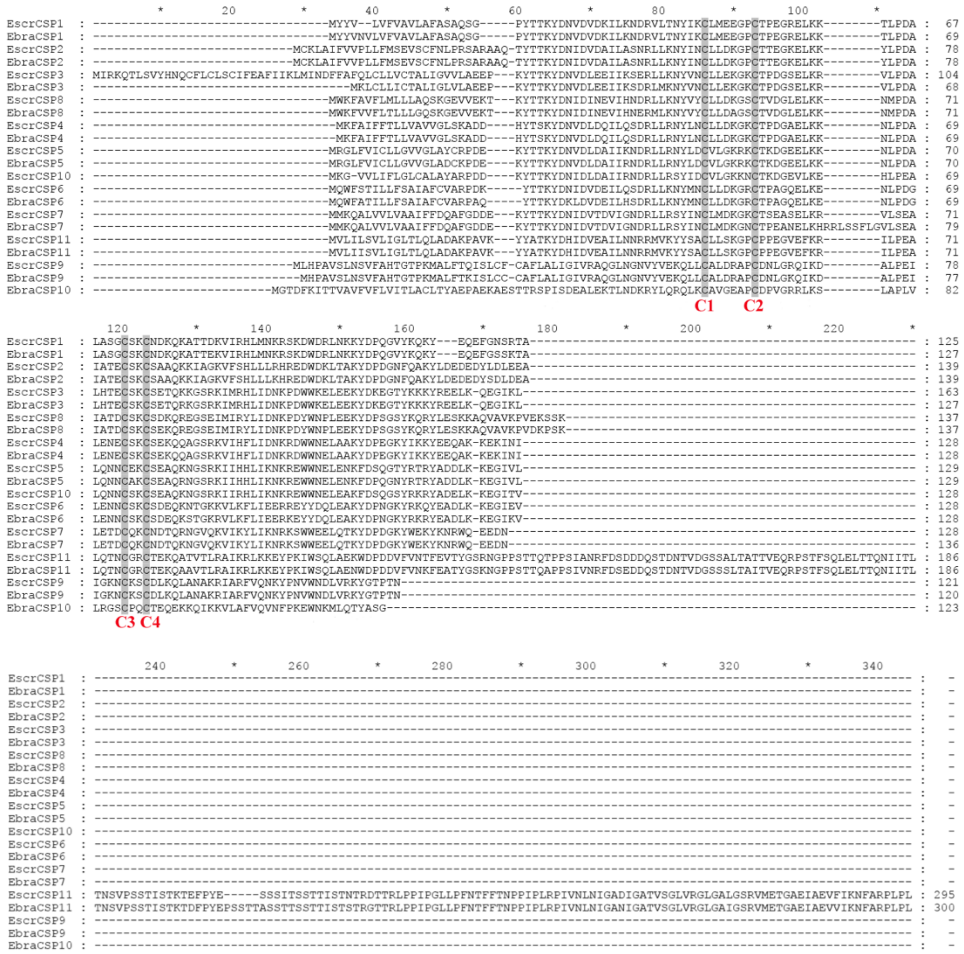

Figure S4 Alignment of amino acid sequences of chemosensory proteins (CSPs) of *E. scrobiculatus* and *E. brandti*. Conserved cysteine sites are marked with red capital letter.

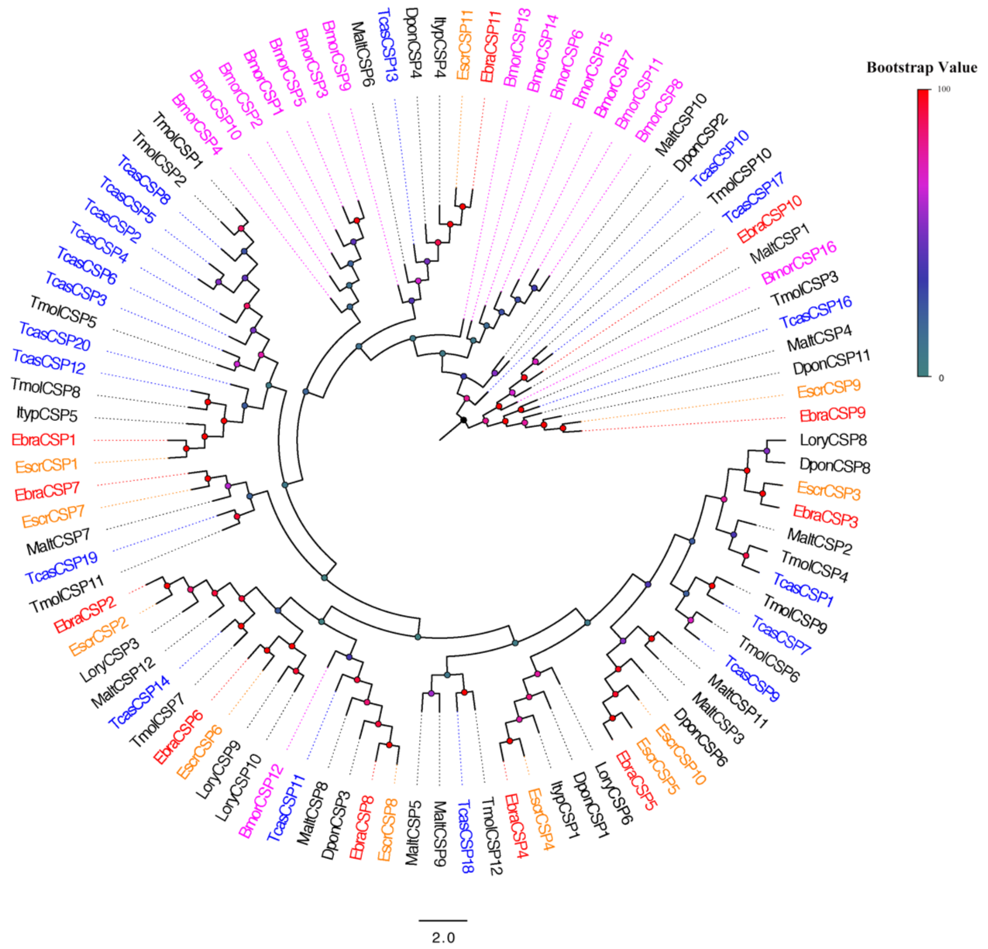

Figure S5 Phylogenetic tree of putative chemosensory protein (CSP) genes. The tree was constructed using MEGA6 with Neighbor-joining method.

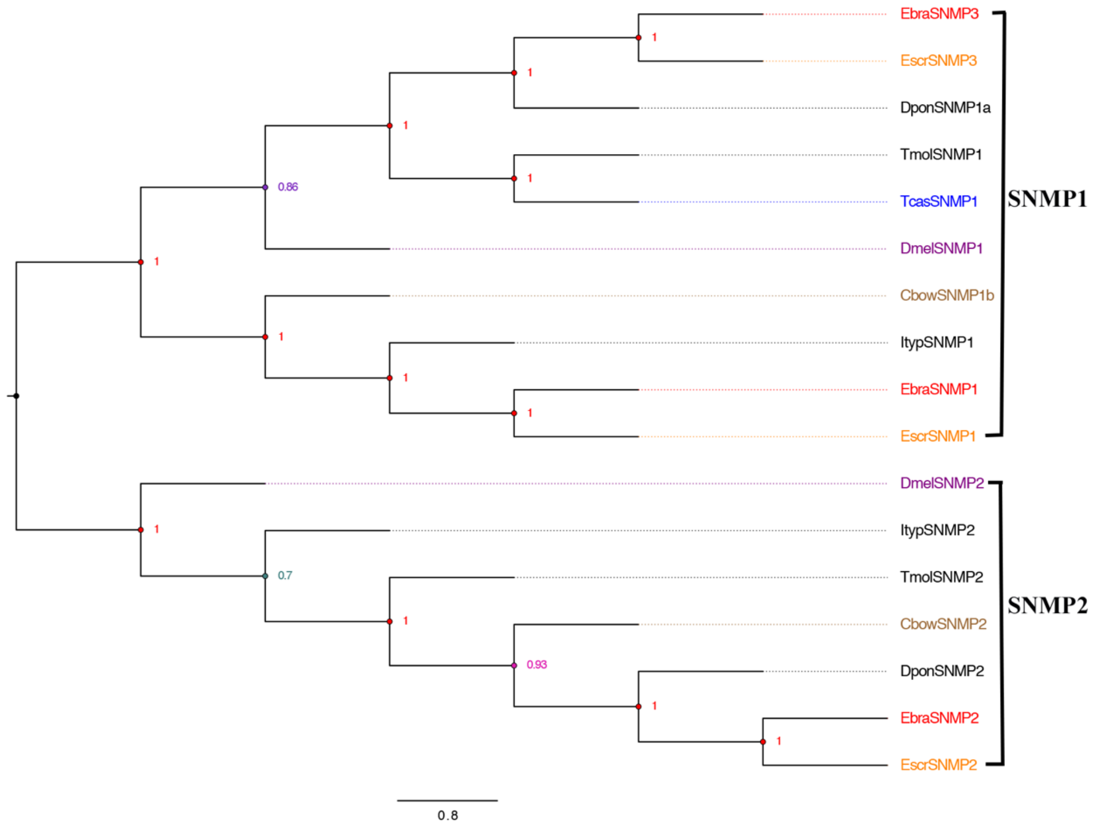

Figure S6 Phylogenetic tree of putative sensory neuron membrane protein (SNMP) genes. The tree was constructed using MEGA6 with Neighbor-joining method.
