# Supplementary material for: Identification and Comparison of Chemosensory Genes in the Antennal Transcriptomes of Eucryptorrhynchus scrobiculatus and E. brandti Fed on Ailanthus altissima
Source: Front Physiol. 2018 Nov 20;9:1652. doi: 10.3389/fphys.2018.01652 (PMC6256065; doi:10.3389/fphys.2018.01652)
Supplement: Supplementary file 2 [file Data_Sheet_2.PDF]

Table S1. An overview of the antennal transcriptome sequencing and assembly of *E. scrobiculatus* and *E. brandti*.

	Clean reads	Q30 (%)	Number of Transcripts	Unigene		
				Number	Mean Length (bp)	N50 (bp)
ESF-1	48,782,922	95.92				
ESF-2	48,439,438	95.99	68316	31757	1437	3064
ESF-3	48,442,962	95.99				
ESM-1	49,858,128	95.91				
ESM-2	48,625,198	95.9	69480	32923	1396	3023
ESM-3	49,504,176	96.01				
ES	--	--	--	46380	3134	4850
EBF-1	41,019,430	96.34				
EBF-2	49,091,354	95.77	86490	44720	1064	1901
EBF-3	49,329,876	95.79				
EBM-1	48,473,014	96.23				
EBM-2	49,375,092	95.84	82384	37712	1212	2195
EBM-3	49,215,254	95.91				
EB	--	--	--	56084	2130	3396

Table S2. The annotation statistics of *E. scrobiculatus* and *E. brandti* unigenes.

	Annotated in BLASTX	Annotated in BLASTP	Annotated in KEGG	Annotated in GO
ESF	10881	11080	11354	11388
ESM	10873	11061	11360	11392
ES	29631	30151	30641	30714
EBF	13797	14085	14428	14473
EBM	11798	12043	12334	12363
EB	26487	27058	27621	27666

Table S3. Best BLASTX matches of candidate odorant binding proteins (OBPs) in *E. scrobiculatus* and *E. brandti*

Gene name	Transcript ID	Unigene Length (bp)	ORF length (AA)	Complete ORF	signal peptide (AA)	Blastx Match						FPKM	
						Name	Species	Score	E-value	Identity	Acc.number	F	M
EscrOBP1	ESF_DN23096_c0_g1_i1	937	176	YES	1-20	odorant binding protein 18	*Colaphellus bowringi*	241.00	1e-76	65%	ALR72506.1	47.21	37.59
EscrOBP2	CL4715Contig1	1682	234	YES	1-18	odorant binding protein 2	*Dendroctonus ponderosae*	202.00	8e-58	53%	AKK25130.1	290.78	273.12
EscrOBP3	CL10113Contig1	764	152	YES	1-19	odorant binding protein	*Lissorhoptrus oryzophilus*	258.00	5e-84	82%	AHE13793.1	1194.01	1115.80
EscrOBP4	CL6644Contig1	1010	143	YES	1-21	odorant binding protein 9	*Rhynchophorus ferrugineus*	222.00	3e-69	71%	ANE37553.1	24.99	30.47
EscrOBP5	CL298Contig2	824	144	YES	1-21	odorant binding protein 16	*Dendroctonus ponderosae*	213.00	7e-67	72%	AKK25140.1	12.40	13.12
EscrOBP6	CL1Contig275	1979	144	YES	1-16	odorant binding protein 8	*Dendroctonus ponderosae*	144.00	4e-36	56%	AGI05175.1	1976.87	2217.87
EscrOBP7	CL9577Contig1	616	146	YES	1-22	odorant binding protein 8	*Dendroctonus armandi*	82.40	2e-16	45%	ALM64970.1	783.07	702.55
EscrOBP8	CL2127Contig1	601	134	YES	1-19	pheromone binding protein 1	*Anoplophora glabripennis*	168.00	3e-50	66%	ASA46120.1	48.03	50.41
EscrOBP9	CL13062Contig1	533	142	YES	1-22	odorant binding protein 8	*Dendroctonus armandi*	82.80	5e-17	42%	ALM64970.1	3.28	2.03
EscrOBP10	CL2963Contig1	2911	133	YES	1-22	odorant binding protein 5	*Dendroctonus armandi*	126.00	3e-30	56%	ALM64967.1	3561.71	3813.92
EscrOBP11	ESF_DN21820_c0_g1_i5	1584	136	YES	1-19	odorant binding protein 19	*Dendroctonus ponderosae*	173.00	2e-48	68%	AKK25143.1	1377.49	1183.58
EscrOBP12	CL6373Contig1	1568	136	YES	1-19	odorant binding protein 19	*Dendroctonus ponderosae*	179.00	1e-50	71%	AKK25143.1	0.16	0.23
EscrOBP13	CL7458Contig1	743	133	YES	1-18	odorant binding protein 1	*Rhynchophorus ferrugineus*	174.00	6e-52	71%	ANE37545.1	2075.88	1976.28
EscrOBP14	ESF_DN17765_c0_g1_i1	990	150	YES	1-21	odorant binding protein 4	*Colaphellus bowringi*	94.00	5e-20	46%	ALR72492.1	0.61	0.31
EscrOBP15	CL1640Contig2	1707	139	YES	1-24	odorant binding protein 2	*Rhynchophorus ferrugineus*	75.50	1e-12	37%	ANE37546.1	493.29	539.43
EscrOBP16	CL13188Contig1	657	133	YES	1-17	odorant binding protein 9	*Dendroctonus ponderosae*	164.00	3e-48	58%	AKK25135.1	485.62	675.53
EscrOBP17	TRINITY_DN21042_c0_g2_i1	958	139	YES	1-19	odorant binding protein 20	*Dendroctonus ponderosae*	156.00	6e-44	55%	AKK25144.1	1.43	0.00
EscrOBP18	ESM_DN21006_c0_g1_i3	825	174	YES	NO	odorant binding protein	*Lissorhoptrus oryzophilus*	163.00	4e-47	51%	AHE13800.1	7744.48	8389.93
EscrOBP19	CL5781Contig1	597	123	YES	1-17	odorant binding protein 6	*Dendroctonus armandi*	100.00	7e-24	40%	ALM64968.1	28.68	32.78
EscrOBP20	TRINITY_DN16252_c0_g1_i1	613	144	YES	1-16	odorant binding protein	*Lissorhoptrus oryzophilus*	143.00	3e-40	52%	AHE13794.1	13.39	15.28
EscrOBP21	CL20968Contig1	1422	149	YES	NO	odorant-binding protein 11	*Tenebrio molitor*	51.20	4e-04	41%	AJM71485.1	83.72	106.42
EscrOBP22	TRINITY_DN15635_c0_g1_i2	701	124	YES	NO	odorant binding protein C03	*Tribolium castaneum*	100.00	5e-23	45%	EFA07546.1	0.27	0.00
EscrOBP23	CL5489Contig1	617	132	YES	1-18	odorant binding protein 19	*Dendroctonus ponderosae*	64.70	9e-10	42%	AGI05183.1	75.98	110.38
EscrOBP24	ESF_DN22291_c1_g6_i4	630	104	YES	1-22	pheromone binding protein 1	*Phyllopertha diversa*	79.70	3e-16	54%	BAC66783.1	3.26	2.27
EscrOBP25	CL6157Contig1	607	133	YES	1-18	odorant binding protein 6	*Rhynchophorus ferrugineus*	103.00	1e-24	40%	ANE37550.1	24.90	27.13
EscrOBP26	CL12686Contig1	453	134	YES	1-16	odorant-binding protein 21	*Dastarcus helophoroides*	84.70	4e-18	37%	AIX97067.1	1.55	1.37
EscrOBP27	CL11150Contig1	739	195	YES	NO	odorant binding protein 4	*Dendroctonus ponderosae*	270.00	1e-88	65%	AGI05167.1	0.48	0.34
EscrOBP28	CL8465Contig1	679	143	YES	1-18	odorant binding protein	*Rhynchophorus ferrugineus*	150.00	2e-42	58%	AMK48596.1	32.54	15.53
EscrOBP29	CL8403Contig1	543	158	YES	1-27	odorant-binding protein 9	*Tenebrio molitor*	186.00	2e-57	55%	AJM71483.1	4.35	4.59
EscrOBP30	CL6649Contig1	1077	159	YES	1-22	odorant binding protein 8	*Dendroctonus armandi*	41.20	0.88	37%	ALM64970.1	1.39	1.57
EscrOBP31	TRINITY_DN22541_c0_g2_i3	522	80	NO	1-20	odorant-binding protein 20	*Pyrrhalta maculicollis*	74.30	8e-14	66%	APC94211.1	0.00	8.61
EbraOBP1	CL17410Contig1	939	176	YES	1-20	odorant binding protein 18	*Colaphellus bowringi*	243	3e-77	66%	ALR72506.1	35.19	35.69
EbraOBP2	CL2658Contig2	811	128	YES	NO	odorant-binding protein 19	*Monochamus alternatus*	135	2e-35	61%	AIX97034.1	753.26	707.64
EbraOBP3	TRINITY_DN26781_c0_g1_i1	895	152	YES	1-19	odorant binding protein	*Lissorhoptrus oryzophilus*	236	4e-75	88%	AHE13793.1	378.56	280.96
EbraOBP4	CL3062Contig2	842	143	YES	1-21	odorant binding protein 9	*Rhynchophorus ferrugineus*	220	2e-69	71%	ANE37553.1	570.90	600.79
EbraOBP5	TRINITY_DN26797_c0_g1_i3	484	122	YES	NO	odorant binding protein 3	*Dendroctonus armandi*	206	7e-66	78%	ALM64965.1	3.34	106.79
EbraOBP6	CL4431Contig1	1666	144	YES	1-16	odorant-binding protein 8	*Dendroctonus ponderosae*	144	2e-36	56%	AGI05175.1	6.98	6.80
EbraOBP7	CL19643Contig1	597	146	YES	1-22	odorant binding protein 8	*Dendroctonus armandi*	82	2e-16	45%	ALM64970.1	2.40	0.83
EbraOBP8	CL49Contig1	2599	111	YES	NO	pheromone binding protein 1	*Anoplophora glabripennis*	165	7e-44	65%	ASA46120.1	6.55	6.79
EbraOBP9	CL6Contig39	830	148	YES	1-22	odorant binding protein 8	*Dendroctonus armandi*	83.2	4e-16	42%	ALM64970.1	494.69	612.25
EbraOBP10	CL21053Contig1	506	133	YES	1-22	odorant binding protein 5	*Dendroctonus armandi*	126	2e-34	56%	ALM64967.1	45.09	46.12
EbraOBP11	CL20153Contig1	578	136	YES	1-19	odorant binding protein 19	*Dendroctonus ponderosae*	171	1e-51	68%	AKK25143.1	7.67	8.21
EbraOBP12	CL2484Contig1	580	136	YES	1-19	odorant binding protein 19	*Dendroctonus ponderosae*	179	8e-55	71%	AKK25143.1	4.21	4.27
EbraOBP13	CL6315Contig1	590	133	YES	1-18	odorant binding protein 14	*Dendroctonus armandi*	194	2e-60	68%	ALM64972.1	971.60	644.78
EbraOBP14	TRINITY_DN36412_c0_g1_i2	567	150	YES	1-21	odorant-binding protein 2	*Dastarcus helophoroides*	88.6	2e-19	46%	AIX97077.1	3.30	1.43
EbraOBP15	CL384Contig6	1257	139	YES	1-21	odorant binding protein 2	*Rhynchophorus ferrugineus*	76.6	3e-13	37%	ANE37546.1	197.10	199.73
EbraOBP16	CL5467Contig1	610	133	YES	1-17	odorant binding protein 9	*Dendroctonus ponderosae*	167	1e-49	60%	AKK25135.1	34.26	39.43
EbraOBP17	CL17648Contig1	816	139	YES	1-19	odorant binding protein 20	*Dendroctonus ponderosae*	155	3e-44	55%	AKK25144.1	1.40	1.33
EbraOBP18	CL374Contig3	951	143	YES	1-20	odorant binding protein	*Lissorhoptrus oryzophilus*	163	2e-46	51%	AHE13800.1	16.31	16.04
EbraOBP19	EBF_DN31064_c0_g2_i3	460	138	YES	1-19	odorant binding protein 3	*Dendroctonus ponderosae*	193	6e-61	63%	AKK25131.1	55.03	49.76
EbraOBP20	CL70Contig7	684	144	YES	1-16	odorant binding protein	*Lissorhoptrus oryzophilus*	137	1e-37	50%	AHE13794.1	3.81	2.68
EbraOBP21	EBF_DN32068_c0_g1_i4	1058	134	YES	1-19	odorant-binding protein 15	*Tenebrio molitor*	62	4e-08	31%	AJM71489.1	6.82	6.20
EbraOBP22	CL9974Contig1	589	160	YES	1-16	odorant binding protein 13	*Dendroctonus armandi*	78.2	6e-15	43%	ALM64971.1	16.79	23.03
EbraOBP23	CL6169Contig1	760	132	YES	1-18	odorant binding protein 18	*Dendroctonus ponderosae*	55.8	3e-06	61%	AKK25142.1	208.59	143.20
EbraOBP24	TRINITY_DN35285_c0_g2_i3	407	116	YES	NO	pheromone binding protein 1	*Anoplophora glabripennis*	134	7e-38	58%	ASA46120.1	0.00	0.00
EbraOBP25	CL384Contig4	2005	132	YES	1-18	odorant binding protein 3	*Rhynchophorus ferrugineus*	102	8e-22	41%	ANE37547.1	9.55	11.43
EbraOBP26	CL11207Contig1	1041	135	YES	1-21	odorant-binding protein 8	*Monochamus alternatus*	50.8	4e-04	39%	AIX97023.1	3.03	2.29
EbraOBP27	CL5427Contig1	1192	147	YES	1-22	odorant binding protein 8	*Dendroctonus armandi*	45.4	0.033	39%	ALM64970.1	0.84	0.66
EbraOBP28	CL49Contig10	3090	119	YES	NO	odorant binding protein	*Lissorhoptrus oryzophilus*	131	4e-32	60%	AHE13796.1	28.02	21.43

Table S4. Best BLASTX matches of candidate chemosensory proteins (CSPs) in *E. scrobiculatus* and *E. brandti*

Gene name	Transcript ID	Unigene Length (bp)	ORF length (AA)	Complete ORF	signal peptide (AA)	Blastx Match						FPKM	
						Name	Species	Score	E-value	Identity	Acc.number	F	M
EscrCSP1	CL8303Contig1	594	125	YES	1-16	chemosensory protein CSP8	*Tenebrio molitor*	163	1e-48	74%	AJO62214.1	4.88	5.24
EscrCSP2	ESF_DN17559_c0_g1_i2	573	138	YES	1-19	chemosensory protein 3	*Lissorhoptrus oryzophilus*	169	1e-50	73%	AHE13801.1	1.05	0.58
EscrCSP3	CL1306Contig1	2945	163	YES	NO	chemosensory protein 8	*Dendroctonus ponderosae*	221	9e-64	82%	AGI05164.1	12.96	11.43
EscrCSP4	CL3009Contig1	749	128	YES	1-19	chemosensory protein 1	*Dendroctonus ponderosae*	179	7e-54	77%	AGI05161.1	5651.73	7188.69
EscrCSP5	CL13088Contig1	521	129	YES	1-18	chemosensory protein	*Anoplophora chinensis*	169	4e-51	67%	AUF73002.1	19.87	19.42
EscrCSP6	CL4595Contig1	551	128	YES	1-18	chemosensory protein 9	*Lissorhoptrus oryzophilus*	176	2e-53	67%	AHE13804.1	1624.19	1723.04
EscrCSP7	CL24Contig5	1000	128	YES	1-20	chemosensory protein 7	*Monochamus alternatus*	139	2e-37	50%	AIX97047.1	46.20	51.57
EscrCSP8	CL771Contig1	545	137	YES	1-17	chemosensory protein 12	*Colaphellus bowringi*	206	5e-65	73%	ALR72526.1	0.00	0.00
EscrCSP9	CL4959Contig1	891	121	YES	NO	chemosensory protein 6	*Dendroctonus ponderosae*	160	3e-46	81%	AKK25149.1	9.88	10.03
EscrCSP10	CL13412Contig1	510	128	YES	1-17	chemosensory protein 5	*Monochamus alternatus*	173	1e-52	60%	AIX97045.1	8.97	9.90
EscrCSP11	CL22340Contig1	2198	295	YES	1-18	chemosensory protein 4	*Dastarcus helophoroides*	172	3e-45	71%	AIX97072.1	7.88	5.34
EbraCSP1	CL7840Contig1	834	127	YES	1-18	chemosensory protein CSP8	*Tenebrio molitor*	164	1e-47	75%	AJO62214.1	0.56	0.66
EbraCSP2	CL3000Contig1	1136	139	YES	1-19	chemosensory protein 3	*Dendroctonus ponderosae*	216	1e-66	72%	AGI05160.1	1087.10	818.48
EbraCSP3	CL8976Contig1	644	127	YES	1-17	chemosensory protein 8	*Dendroctonus ponderosae*	226	4e-73	84%	AGI05164.1	5712.04	4332.92
EbraCSP4	CL21195Contig1	750	128	YES	1-19	chemosensory protein 1	*Dendroctonus ponderosae*	179	7e-54	77%	AGI05161.1	18.62	17.69
EbraCSP5	CL12947Contig1	581	129	YES	1-18	chemosensory protein	*Anoplophora chinensis*	168	2e-50	66%	AUF73002.1	1.64	1.94
EbraCSP6	CL9072Contig1	730	128	YES	1-18	chemosensory protein 9	*Lissorhoptrus oryzophilus*	169	6e-50	63%	AHE13804.1	2397.71	1850.24
EbraCSP7	EBM_DN27580_c0_g1_i3	622	136	NO	1-20	chemosensory protein 2	*Monochamus alternatus*	126	2e-33	54%	AIX97042.1	3.91	4.04
EbraCSP8	TRINITY_DN31530_c0_g1_i1	1394	137	YES	1-17	chemosensory protein 3	*Dendroctonus ponderosae*	213	4e-64	71%	AGI05160.1	4.23	0.66
EbraCSP9	CL20684Contig1	560	120	YES	NO	chemosensory protein 6	*Dendroctonus ponderosae*	154	3e-45	78%	AKK25149.1	99.19	105.04
EbraCSP10	CL17628Contig1	948	123	YES	1-26	chemosensory protein 11	*Colaphellus bowringi*	142	1e-38	76%	ALR72525.1	9.11	9.04
EbraCSP11	CL18115Contig1	2496	300	YES	1-18	chemosensory protein 2	*Phyllotreta striolata*	167	9e-43	66%	ANQ46492.1	3.56	3.89

Table S5. Best BLASTX matches of candidate odorant receptors (OR) in *E. scrobiculatus* and *E. brandti*

Gene name	Transcript ID	Unigene Length (bp)	ORF length (AA)	Complete ORF	TMD	Blastx Match						FPKM	
						Name	Species	Score	E-value	Identity	Acc.number	F	M
EscrOR1	CL23257Contig1	1534	382	YES	7	odorant receptor 8	*Pyrrhalta maculicollis*	228	4e-66	36%	APC94235.1	9.88	10.46
EscrOR2	CL9068Contig1	1321	383	YES	4	odorant receptor 41	*Anoplophora chinensis*	213	3e-61	36%	AVN97853.1	2.99	2.83
EscrOR3	CL22121Contig1	1970	429	YES	7	odorant receptor OR36	*Colaphellus bowringi*	369	8e-119	50%	ALR72579.1	1.71	1.62
EscrOR4	ESM_DN19912_c0_g2_i2	1901	420	YES	7	odorant receptor OR11	*Colaphellus bowringi*	404	7e-134	60%	ALR72556.1	1.19	0.55
EscrOR5	CL19355Contig1	1240	381	YES	5	odorant receptor 48	*Anoplophora chinensis*	180	8e-49	33%	AVN97860.1	4.63	3.26
EscrOR6	CL4879Contig1	2012	327	YES	6	odorant receptor 9	*Anoplophora chinensis*	58.2	9e-07	42%	AVN97821.1	1.80	1.83
EscrOR7	CL20062Contig1	1340	377	YES	6	olfactory receptor 3	*Rhyzopertha dominica*	91.7	8e-18	33%	AIX97138.1	2.45	1.99
EscrOR8	CL4360Contig1	2054	391	YES	4	odorant receptor 15	*Dendroctonus ponderosae*	163	1e-40	30%	AKK25156.1	2.81	2.91
EscrOR9	CL13321Contig1	1631	380	YES	3	odorant receptor OR20	*Colaphellus bowringi*	112	2e-24	30%	ALR72565.1	3.81	4.40
EscrOR10	CL15782Contig1	2435	392	YES	5	odorant receptor 21	*Anoplophora chinensis*	75.9	1e-12	37%	AVN97833.1	5.13	4.41
EscrOR11	CL20301Contig1	1368	395	YES	5	odorant receptor 28	*Anoplophora chinensis*	85.5	1e-16	45%	AVN97840.1	9.47	8.95
EscrOR12	CL19979Contig1	1725	385	YES	6	odorant receptor 5	*Dendroctonus ponderosae*	210	2e-61	55%	AKK25153.1	1.84	1.78
EscrOR13	CL19279Contig1	1284	399	YES	3	odorant receptor 28	*Anoplophora chinensis*	69.7	4e-11	45%	AVN97840.1	3.99	3.71
EscrOR14	CL19579Contig1	1206	384	NO	4	odorant receptor 9	*Anoplophora chinensis*	73.6	2e-12	43%	AVN97821.1	3.28	3.24
EscrOR15	CL23781Contig1	1659	406	YES	1	odorant receptor 28	*Anoplophora chinensis*	63.2	1e-08	37%	AVN97840.1	4.94	4.30
EscrOR16	CL19883Contig1	1555	407	YES	4	odorant receptor 14	*Anoplophora chinensis*	100	2e-21	38%	AVN97826.1	2.29	1.72
EscrOR17	CL20297Contig1	1630	407	YES	8	odorant receptor 9	*Anoplophora chinensis*	102	1e-22	48%	AVN97821.1	11.69	11.15
EscrOR18	CL20392Contig1	1410	415	YES	7	odorant receptor 14	*Anoplophora chinensis*	88.6	4e-17	35%	AVN97826.1	4.37	3.80
EscrOR19	CL22934Contig1	1613	402	YES	7	odorant receptor 21	*Anoplophora chinensis*	104	4e-23	42%	AVN97833.1	5.41	6.85
EscrOR20	CL19886Contig1	1602	411	YES	5	odorant receptor 10	*Anoplophora chinensis*	182	2e-48	31%	AVN97822.1	14.66	12.72
EscrOR21	CL809Contig1	2862	387	YES	6	odorant receptor OR6	*Colaphellus bowringi*	214	5e-58	32%	ALR72551.1	2.87	3.24
EscrOR22	CL19464Contig1	1339	396	YES	7	odorant receptor 73	*Tribolium castaneum*	162	3e-40	30%	EFA05710.2	5.72	4.76
EscrOR23	CL21067Contig1	1268	393	YES	7	odorant receptor 73	*Tribolium castaneum*	392	6e-128	54%	EFA05710.2	2.52	2.90
EscrOR24	ESF_DN22058_c0_g1_i2	2034	482	YES	7	olfactory co-receptor	*Rhynchophorus ferrugineus*	870	0.0	92%	AOO35283.1	0.12	0.06
EscrOR25	CL23284Contig1	1760	397	YES	7	odorant receptor 295	*Tribolium castaneum*	63.2	8e-07	26%	EFA11864.1	1.31	2.29
EscrOR26	CL19438Contig1	1327	399	YES	6	olfactory receptor 3	*Rhyzopertha dominica*	86.3	7e-16	33%	AIX97138.1	2.28	2.76
EscrOR27	CL23324Contig1	1494	404	YES	5	odorant receptor 28	*Anoplophora chinensis*	82.4	2e-15	42%	AVN97840.1	13.98	13.39
EscrOR28	CL22116Contig1	1953	389	YES	5	odorant receptor 5	*Dendroctonus ponderosae*	104	5e-22	33%	AKK25153.1	4.06	3.80
EscrOR29	CL802Contig1	1793	388	YES	6	odorant receptor 2	*Anoplophora chinensis*	82.8	4e-15	37%	AVN97814.1	1.04	1.14
EscrOR30	CL20437Contig1	1398	388	YES	6	olfactory receptor OR16	*Tenebrio molitor*	165	9e-43	35%	AJO62235.1	3.78	2.81
EscrOR31	CL19512Contig1	1286	400	YES	7	odorant receptor 24	*Dendroctonus ponderosae*	393	1e-131	50%	AGI05166.1	25.81	21.57
EscrOR32	CL21503Contig1	1194	390	NO	4	odorant receptor 7	*Dendroctonus ponderosae*	113	3e-27	70%	AKK25154.1	2.23	1.78
EscrOR33	CL3640Contig1	2119	392	YES	4	odorant receptor 7	*Dendroctonus ponderosae*	72.4	9e-12	42%	AKK25154.1	0.66	0.59
EscrOR34	ESF_DN18114_c0_g1_i3	1466	329	YES	6	olfactory receptor OR13	*Tenebrio molitor*	92.8	6e-18	33%	AJO62232.1	11.47	10.29
EscrOR35	CL14596Contig1	2809	387	YES	7	odorant receptor 20	*Anoplophora chinensis*	89	3e-17	36%	AVN97832.1	1.45	1.17
EscrOR36	CL22509Contig1	1466	388	YES	7	odorant receptor 19	*Pyrrhalta aenescens*	158	4e-40	28%	APC94313.1	4.15	3.91

EscrOR37	CL5681Contig1	1371	401	YES	7	odorant receptor	*Anoplophora chinensis*	82.4	1e-15	49%	AUF73036.1	4.77	4.89
EscrOR38	CL19998Contig1	1649	396	YES	7	odorant receptor 102	*Tribolium castaneum*	198	1e-54	34%	EEZ97750.2	2.46	1.86
EscrOR39	CL21217Contig1	1213	264	YES	5	odorant receptor 309	*Tribolium castaneum*	89.4	7e-16	30%	EEZ97786.1	5.09	5.01
EscrOR40	CL20226Contig1	1325	389	YES	7	odorant receptor OR21	*Colaphellus bowringi*	123	7e-28	41%	ALR72566.1	5.54	4.52
EscrOR41	CL20342Contig1	1433	427	YES	5	odorant receptor OR39	*Colaphellus bowringi*	82.4	3e-15	43%	ALR72582.1	3.40	2.74
EscrOR42	CL8552Contig1	906	183	YES	2	odorant receptor 7	*Dendroctonus ponderosae*	75.9	9e-14	40%	AKK25154.1	12.33	13.65
EscrOR43	CL15117Contig1	2697	417	YES	4	odorant receptor 20	*Anoplophora chinensis*	103	2e-22	42%	AVN97832.1	11.77	11.20
EscrOR44	CL5443Contig1	521	132	YES	2	odorant receptor 7	*Dendroctonus ponderosae*	125	3e-34	76%	AKK25154.1	4.64	4.51
EscrOR45	CL3838Contig1	2745	377	YES	4	odorant receptor 7	*Dendroctonus ponderosae*	80.1	2e-14	43%	AKK25154.1	3.84	3.64
EscrOR46	CL14945Contig1	2679	392	YES	5	odorant receptor 28	*Anoplophora chinensis*	76.3	5e-13	40%	AVN97840.1	4.10	3.95
EscrOR47	CL21421Contig1	1182	367	NO	6	odorant receptor 10	*Anopheles stephensi*	79.7	1e-12	31%	ACH69150.1	2.24	2.10
EscrOR48	CL1889Contig1	1546	404	YES	5	odorant receptor 46	*Microplitis mediator*	69.3	7e-09	30%	AKO90010.1	2.05	2.08
EscrOR49	CL2210Contig1	2387	386	NO	7	odorant receptor	*Anoplophora chinensis*	119	2e-27	38%	AUF73017.1	0.75	0.54
EbraOR1	CL7833Contig1	846	192	YES	3	odorant receptor 8	*Pyrrhalta maculicollis*	203	2e-59	46%	APC94235.1	6.58	15.36
EbraOR2	CL6406Contig1	1416	383	YES	6	odorant receptor 41	*Anoplophora chinensis*	220	8e-64	35%	AVN97853.1	3.33	4.17
EbraOR3	CL18786Contig1	1171	356	YES	6	odorant receptor 8	*Anoplophora chinensis*	261	8e-83	57%	AVN97820.1	15.39	14.32
EbraOR4	CL2166Contig1	3334	419	YES	7	odorant receptor OR11	*Colaphellus bowringi*	410	1e-130	59%	ALR72556.1	0.21	0.30
EbraOR5	CL2141Contig2	1285	365	YES	6	olfactory receptor OR7	*Tenebrio molitor*	133	5e-31	33%	AJO62226.1	0.26	0.39
EbraOR6	CL18081Contig1	1339	393	YES	6	odorant receptor 15	*Dendroctonus ponderosae*	177	3e-47	30%	AKK25156.1	4.08	5.17
EbraOR7	CL5971Contig1	1034	326	NO	6	odorant receptor 15	*Dendroctonus ponderosae*	108	7e-23	28%	AKK25156.1	3.72	5.80
EbraOR8	CL16331Contig1	1180	354	YES	4	odorant receptor OR20	*Colaphellus bowringi*	86.3	1e-15	29%	ALR72565.1	3.37	3.81
EbraOR9	EBM_DN27217_c0_g1_i3	1197	323	YES	5	odorant receptor OR20	*Colaphellus bowringi*	111	1e-24	28%	ALR72565.1	50.51	47.23
EbraOR10	CL870Contig4	1487	225	YES	3	odorant receptor 15	*Dendroctonus ponderosae*	149	3e-36	37%	AKK25156.1	11.02	11.37
EbraOR11	CL3165Contig1	1336	395	YES	6	odorant receptor 5	*Dendroctonus ponderosae*	124	6e-30	34%	AKK25153.1	5.28	6.69
EbraOR12	CL9703Contig1	1310	385	YES	6	odorant receptor 5	*Dendroctonus ponderosae*	218	6e-66	58%	AKK25153.1	4.18	4.94
EbraOR13	CL18123Contig1	1308	399	YES	3	odorant receptor OR20	*Colaphellus bowringi*	89.7	1e-16	31%	ALR72565.1	7.21	8.20
EbraOR14	CL18003Contig1	1287	387	YES	5	odorant receptor 15	*Dendroctonus ponderosae*	122	2e-27	27%	AKK25156.1	8.95	10.17
EbraOR15	CL22161Contig1	1670	408	YES	6	odorant receptor 28	*Anoplophora chinensis*	64.7	3e-09	35%	AVN97840.1	3.72	4.39
EbraOR16	CL2568Contig1	1479	407	YES	4	odorant receptor 14	*Anoplophora chinensis*	103	3e-22	38%	AVN97826.1	2.98	2.72
EbraOR17	CL839Contig1	1402	407	YES	8	odorant receptor 9	*Anoplophora chinensis*	101	2e-22	52%	AVN97821.1	4.79	4.52
EbraOR18	CL4151Contig1	1460	415	YES	8	odorant receptor 14	*Anoplophora chinensis*	76.3	7e-13	37%	AVN97826.1	12.15	12.59
EbraOR19	CL10465Contig1	1195	279	YES	5	odorant receptor 21	*Anoplophora chinensis*	105	1e-23	42%	AVN97833.1	6.99	7.79
EbraOR20	CL10838Contig1	1258	333	YES	3	odorant receptor 2	*Anopheles quadriannulatus*	110	3e-23	32%	ACH69144.1	15.51	15.07
EbraOR21	TRINITY_DN23093_c0_g1_i1	1293	404	YES	6	odorant receptor 14	*Anoplophora chinensis*	81.3	1e-14	34%	AVN97826.1	48.35	40.67
EbraOR22	CL17724Contig1	1398	396	YES	7	odorant receptor 73	*Tribolium castaneum*	168	4e-42	31%	EFA05710.2	8.03	10.60
EbraOR23	CL18098Contig1	1402	393	YES	7	odorant receptor 73	*Tribolium castaneum*	391	6e-127	53%	EFA05710.2	7.43	7.08
EbraOR24	CL79Contig14	2631	482	YES	7	olfactory co-receptor	*Rhynchophorus ferrugineus*	868	0.0	92%	AOO35283.1	168.24	143.53
EbraOR25	CL19682Contig1	1506	392	YES	7	odorant receptor 222	*Tribolium castaneum*	56.6	7e-05	31%	EFA01407.1	1.50	3.14
EbraOR26	CL7707Contig1	1351	399	YES	6	odorant receptor 10	*Anoplophora chinensis*	146	2e-35	28%	AVN97822.1	6.86	8.64
EbraOR27	CL20070Contig1	1695	404	YES	5	odorant receptor 7	*Dendroctonus ponderosae*	84.3	4e-16	46%	AKK25154.1	11.07	11.04
EbraOR28	CL6591Contig1	1216	338	YES	2	odorant receptor 2	*Anoplophora chinensis*	98.6	3e-21	39%	AVN97814.1	10.58	10.20
EbraOR29	CL355Contig8	1185	338	YES	4	odorant receptor 25	*Anoplophora chinensis*	80.5	9e-15	36%	AVN97837.1	1.55	1.13
EbraOR30	CL17996Contig1	1505	391	YES	6	olfactory receptor OR16	*Tenebrio molitor*	154	3e-38	33%	AJO62235.1	1.35	1.22
EbraOR31	CL6032Contig1	707	224	NO	4	odorant receptor 24	*Dendroctonus ponderosae*	173	1e-48	44%	AGI05166.1	19.36	20.63
EbraOR32	CL10569Contig1	1435	398	YES	5	odorant receptor 7	*Dendroctonus ponderosae*	133	3e-34	76%	AKK25154.1	5.31	7.95
EbraOR33	EBF_DN34813_c0_g2_i6	1483	296	YES	3	odorant receptor 2	*Anoplophora chinensis*	68.6	3e-10	42%	AVN97814.1	0.44	0.80
EbraOR34	CL10227Contig1	2499	402	YES	6	odorant receptor	*Anoplophora chinensis*	124	1e-28	34%	AUF73038.1	5.41	5.21
EbraOR35	CL10362Contig1	1842	387	YES	7	odorant receptor OR6	*Colaphellus bowringi*	215	2e-60	33%	ALR72551.1	1.62	2.45
EbraOR36	CL2701Contig1	1452	401	YES	7	odorant receptor 6	*Pyrrhalta aenescens*	167	4e-43	32%	APC94312.1	3.26	4.30
EbraOR37	CL6485Contig1	891	204	YES	4	odorant receptor 73	*Tribolium castaneum*	119	1e-26	30%	EFA05710.2	7.11	5.65
EbraOR38	CL12385Contig1	1433	396	YES	7	odorant receptor 13	*Cnaphalocrocis medinalis*	76.6	9e-13	46%	ALT31667.1	3.20	8.15
EbraOR39	CL18360Contig1	1956	393	YES	7	odorant receptor 243	*Tribolium castaneum*	55.8	2e-04	30%	EFA12943.1	4.93	8.75
EbraOR40	CL19325Contig1	1365	378	YES	6	olfactory receptor 33	*Helicoverpa assulta*	56.2	3e-05	39%	AJD81568.1	1.20	1.25
EbraOR41	CL15604Contig1	585	182	YES	1	odorant receptor 3	*Drosicha corpulenta*	42.4	0.15	34%	ALV87618.1	1.21	1.20
EbraOR42	CL22445Contig1	944	206	YES	2	olfactory receptor OR16	*Tenebrio molitor*	112	2e-24	43%	AJO62235.1	1.48	1.38
EbraOR43	CL5979Contig1	1552	374	YES	4	odorant receptor 10	*Aedes aegypti*	108	5e-22	32%	ACH69136.1	1.41	1.84
EbraOR44	CL840Contig1	1687	396	YES	4	odorant receptor 7	*Dendroctonus ponderosae*	100	7e-22	55%	AKK25154.1	1.60	1.40
EbraOR45	CL1772Contig1	1793	385	YES	7	odorant receptor 15	*Dendroctonus ponderosae*	166	5e-42	31%	AKK25156.1	5.96	5.60

Table S6. Best BLASTX matches of candidate ionotropic receptors (IR) in *E. scrobiculatus* and *E. brandti*

Gene name	Transcript ID	Unigene Length (bp)	ORF length (AA)	Complete ORF	TMD	Blastx Match						FPKM	
						Name	Species	Score	E-value	Identity	Acc.number	F	M
EscrIR1	CL1Contig134	2996	864	YES	3	ionotropic receptor 93a isoform X2	*Anoplophora glabripennis*	1100	0.0	62%	XP_018576793.1	23.32	25.25
EscrIR2	ESF_DN17175_c0_g1_i2	1510	435	NO	3	ionotropic receptor 40a	*Anoplophora glabripennis*	664	0.0	72%	XP_023310509.1	0.51	0.43
EscrIR3	CL22209Contig1	2441	632	YES	4	ionotropic receptor 6	*Pyrrhalta aenescens*	420	2e-135	44%	APC94350.1	2.63	2.79
EscrIR4	CL17382Contig1	4225	794	YES	3	ionotropic receptor 4	*Pyrrhalta aenescens*	639	0.0	57%	APC94354.1	4.17	3.46

EscrIR5	CL212Contig2	4898	885	YES	3	ionotropic receptor 8a	*Dendroctonus ponderosae*	1343	0.0	74%	AGI05169.1	0.13	0.09
EscrIR6	CL18835Contig1	3839	993	YES	3	ionotropic receptor 3	*Anoplophora chinensis*	1342	0.0	75%	AVN97885.1	45.31	42.11
EscrIR7	CL16617Contig1	2750	625	YES	3	ionotropic receptor 41a	*Colaphellus bowringi*	289	3e-86	46%	ALR72539.1	2.64	3.25
EscrIR8	ESM_DN17718_c0_g1_i7	2505	750	YES	3	ionotropic receptor	*Anoplophora chinensis*	720	0.0	66%	AUF73080.1	0.19	0.29
EscrIR9	CL1Contig5	8772	862	NO	3	glutamate receptor ionotropic, kainate 2	*Anoplophora glabripennis*	1551	0.0	93%	XP_018569892.1	0.87	0.84
EscrIR10	ESF_DN16801_c1_g1_i1	1867	433	YES	4	ionotropic receptor 6	*Pyrrhalta aenescens*	154	5e-37	32%	APC94350.1	0.24	0.19
EscrIR11	ESF_DN23181_c1_g5_i1	2268	480	YES	3	ionotropic receptor 13	*Holotrichia parallela*	303	7e-91	36%	AVH87301.1	0.39	0.35
EscrIR12	CL384Contig1	4710	612	YES	5	ionotropic receptor IR2	*Colaphellus bowringi*	611	0.0	57%	ALR72541.1	0.38	0.38
EscrIR13	CL384Contig8	4223	608	YES	4	chemosensory ionotropic receptor IR2	*Tenebrio molitor*	574	0.0	56%	AJO62240.1	5.04	6.16
EscrIR14	ESF_DN19079_c0_g1_i1	4268	1104	YES	5	Glutamate receptor, ionotropic kainate 2	*Habropoda laboriosa*	947	0.0	69%	KOC70073.1	0.58	0.13
EscrIR15	CL21891Contig1	2003	625	YES	3	ionotropic receptor 6	*Pyrrhalta aenescens*	276	2e-81	36%	APC94350.1	2.02	2.02
EscrIR16	CL7761Contig1	1303	263	YES	1	ionotropic receptor	*Anoplophora chinensis*	208	1e-55	56%	AUF73078.1	1.13	0.56
EscrIR17	CL20866Contig1	785	117	YES	1	ionotropic receptor 12	*Holotrichia parallela*	153	4e-40	43%	AVH87300.1	0.47	0.34
EbraIR1	CL5789Contig1	4475	864	YES	3	ionotropic receptor 14	*Holotrichia parallela*	853	0.0	51%	AVH87302.1	8.08	9.27
EbraIR2	CL7754Contig1	2465	420	YES	1	ionotropic receptor 40a	*Anoplophora glabripennis*	495	0.0	72%	XP_023310509.1	0.89	0.84
EbraIR3	CL11073Contig1	2008	632	YES	4	ionotropic receptor 6	*Pyrrhalta aenescens*	423	3e-138	42%	APC94350.1	1.73	1.73
EbraIR4	CL7637Contig1	4851	792	YES	3	ionotropic receptor 4	*Pyrrhalta aenescens*	639	0.0	57%	APC94354.1	3.32	3.33
EbraIR5	EBF_DN34529_c0_g1_i1	2581	810	YES	2	ionotropic receptor 8a	*Dendroctonus ponderosae*	1230	0.0	75%	AGI05169.1	22.52	22.80
EbraIR6	CL20237Contig1	3286	934	YES	3	ionotropic receptor 3	*Anoplophora chinensis*	1318	0.0	76%	AVN97885.1	73.38	77.29
EbraIR7	CL18209Contig1	2578	616	YES	3	ionotropic receptor 41a	*Colaphellus bowringi*	295	7e-89	48%	ALR72539.1	1.94	1.83
EbraIR8	CL11621Contig1	2750	877	YES	3	glutamate receptor ionotropic, kainate 2 isoform X1	*Aedes aegypti*	795	0.0	48%	XP_021697648.1	0.86	1.29
EbraIR9	CL1Contig1577	4777	861	YES	4	glutamate receptor ionotropic, kainate 2	*Anoplophora glabripennis*	1557	0.0	94%	XP_018569892.1	0.05	0.14
EbraIR10	CL14178Contig1	2604	639	YES	4	ionotropic receptor 6	*Pyrrhalta aenescens*	165	7e-40	30%	APC94350.1	2.91	2.78
EbraIR11	CL6538Contig1	2226	452	YES	3	ionotropic receptor 13	*Holotrichia parallela*	309	2e-93	37%	AVH87301.1	0.59	0.69
EbraIR12	CL5819Contig1	4121	612	YES	4	ionotropic receptor IR2	*Colaphellus bowringi*	622	0.0	57%	ALR72541.1	9.37	8.98
EbraIR13	CL9268Contig1	4276	609	YES	5	chemosensory ionotropic receptor IR2	*Tenebrio molitor*	573	0.0	56%	AJO62240.1	1.57	1.76
EbraIR14	EBF_DN30502_c0_g1_i1	1556	486	YES	3	ionotropic receptor 7	*Phyllotreta striolata*	761	0.0	89%	ANQ46499.1	0.55	0.43
EbraIR15	CL9526Contig1	2029	626	YES	5	ionotropic receptor 6	*Pyrrhalta aenescens*	286	3e-85	37%	APC94350.1	1.78	2.14
EbraIR16	CL7429Contig1	788	196	YES	1	chemosensory ionotropic receptor 75x	*Anomala corpulenta*	144	7e-36	36%	AKC58588.1	5.61	6.93
EbraIR17	CL18846Contig1	2028	625	YES	3	ionotropic receptor 3	*Phyllotreta striolata*	364	7e-115	41%	ANQ46495.1	23.57	36.45
EbraIR18	CL22506Contig1	2990	758	YES	5	chemosensory ionotropic receptor IR4	*Tenebrio molitor*	677	0.0	62%	AJO62242.1	2.11	3.06
EbraIR19	CL18843Contig1	2059	245	YES	2	ionotropic receptor 60a1b	*Heliconius telesiphe sotericus*	46.6	0.19	25%	AMM70737.1	0.40	0.72
EbraIR20	CL17418Contig1	1684	425	YES	2	Glutamate [NMDA] receptor subunit 3A	*Melipona quadrifasciata*	574	0.0	79%	KOX75577.1	0.16	0.13
EbraIR21	CL5675Contig1	2135	668	YES	2	ionotropic receptor 7	*Pyrrhalta aenescens*	779	0.0	54%	APC94352.1	1.17	1.54
EbraIR22	CL450Contig5	2076	628	YES	2	ionotropic receptor	*Anoplophora chinensis*	465	4e-149	42%	AUF73078.1	1.28	1.89
EbraIR23	CL5469Contig1	2271	523	YES	4	ionotropic receptor 6	*Holotrichia parallela*	815	0.0	84%	AVH87294.1	0.45	0.37
EbraIR24	CL21265Contig1	1350	419	YES	3	glutamate receptor 1	*Leptinotarsa decemlineata*	796	0.0	85%	XP_023012213.1	0.36	0.47
EbraIR25	CL285Contig3	2506	627	YES	5	ionotropic receptor 13	*Holotrichia parallela*	338	2e-103	37%	AVH87301.1	12.16	13.02

Table S7. Best BLASTX matches of candidate gustatory receptors (GR) and sensory neuron membrane proteins (SNMP) in *E. scrobiculatus* and *E. brandti*

Gene name	Transcript ID	Unigene Length (bp)	ORF length (AA)	Complete ORF	TMD	Blastx Match						FPKM	
						Name	Species	Score	E-value	Identity	Acc.number	F	M
EscrGR1	CL5421Contig1	2012	456	YES	6	antennal gustatory receptor 8	*Dendrolimus punctatus*	238	1e-70	67%	ARO70280.1	2.02	2.62
EscrGR2	CL16162Contig1	2236	437	YES	7	gustatory receptor 9	*Anoplophora chinensis*	290	4e-87	39%	AVN97874.1	1.41	0.97
EscrGR3	CL3714Contig1	2659	412	YES	7	gustatory receptor 8	*Anoplophora chinensis*	38.9	5.0	29%	AVN97873.1	1.27	0.93
EscrGR4	CL22497Contig1	1490	405	YES	7	gustatory receptor 13	*Pyrrhalta aenescens*	66.2	2e-09	41%	APC94340.1	20.10	22.82
EscrGR5	CL9713Contig1	855	242	YES	3	gustatory receptor 8	*Anoplophora chinensis*	45.4	0.006	37%	AVN97873.1	1.80	1.77
EscrGR6	ESF_DN22744_c2_g1_i3	1531	442	YES	7	gustatory receptor 5	*Colaphellus bowringi*	71.6	5e-12	46%	ALR72531.1	0.61	0.27
EscrGR7	CL6027Contig1	1489	440	YES	6	gustatory receptor 1	*Tribolium castaneum*	598	0.0	73%	EFA07594.2	0.78	0.51
EscrGR8	CL4680Contig1	1469	416	YES	8	gustatory receptor 8	*Pyrrhalta aenescens*	197	2e-56	41%	APC94346.1	0.87	1.04
EscrGR9	CL7280Contig1	1967	407	YES	6	gustatory receptor 17	*Anoplophora chinensis*	45.4	0.18	28%	AVN97882.1	0.48	0.69
EscrGR10	CL12446Contig1	910	228	YES	4	gustatory receptor 2	*Anoplophora chinensis*	119	1e-28	36%	AVN97867.1	1.08	0.77
EscrGR11	CL22844Contig1	1640	390	YES	8	gustatory receptor 12	*Tribolium castaneum*	136	2e-31	30%	EFA04718.1	3.41	4.53
EscrGR12	CL7418Contig1	1216	350	YES	7	gustatory receptor 10	*Holotrichia parallela*	55.8	2e-06	38%	AVH87325.1	1.01	1.01
EscrGR13	CL1Contig1729	1513	325	YES	7	gustatory receptor 7	*Pyrrhalta maculicollis*	193	8e-56	54%	APC94254.1	0.03	0.00
EscrGR14	CL7890Contig1	868	222	YES	3	gustatory receptor 8	*Anoplophora chinensis*	51.2	6e-05	34%	AVN97873.1	0.30	0.51
EscrGR15	CL454Contig2	2913	250	YES	5	gustatory receptor 3	*Anoplophora chinensis*	77	2e-13	48%	AVN97868.1	0.73	0.63
EscrGR16	CL11465Contig1	1088	125	YES	2	gustatory receptor 3	*Pyrrhalta aenescens*	48.1	0.021	41%	APC94333.1	0.24	0.59
EscrGR17	CL1Contig1725	2684	104	YES	1	gustatory receptor 7	*Pyrrhalta aenescens*	42	4.9	42%	APC94345.1	0.97	1.01
EscrGR18	CL10246Contig1	499	58	YES	1	gustatory receptor 15	*Nasonia vitripennis*	48.5	0.002	38%	NP_001177441.1	0.78	0.77
EscrGR19	CL12819Contig1	456	NO	NO	0	gustatory receptor 9	*Anoplophora chinensis*	61.6	3e-08	62%	AVN97874.1	4.35	5.70
EbraGR1	CL5769Contig1	1995	457	YES	6	gustatory receptor 3	*Tribolium castaneum*	465	1e-153	60%	EFA04709.2	5.79	5.63
EbraGR2	CL5153Contig1	2956	426	YES	7	gustatory receptor 9	*Anoplophora chinensis*	293	2e-86	39%	AVN97874.1	1.22	1.60
EbraGR3	CL19269Contig1	1530	412	YES	7	gustatory receptor 5	*Anoplophora chinensis*	49.3	5e-04	36%	AVN97870.1	1.14	1.84

EbraGR4	CL17910Contig1	1446	405	YES	7	gustatory receptor 13	*Pyrrhalta aenescens*	67.4	8e-10	41%	APC94340.1	2.04	1.95
EbraGR5	CL18679Contig1	1534	413	YES	7	gustatory receptor 8	*Anoplophora chinensis*	41.6	0.25	43%	AVN97873.1	1.94	2.34
EbraGR6	CL6060Contig1	2672	438	NO	6	gustatory receptor 5	*Colaphellus bowringi*	70.9	2e-11	46%	ALR72531.1	0.70	1.07
EbraGR7	TRINITY_DN29026_c0_g1_i3	1946	423	YES	4	gustatory receptor 5	*Colaphellus bowringi*	60.1	9e-08	47%	ALR72531.1	2.06	2.53
EbraGR8	CL18385Contig1	2460	396	YES	8	gustatory receptor 14	*Pyrrhalta aenescens*	94.4	1e-16	27%	APC94341.1	3.82	5.32
EbraGR9	CL16487Contig1	1203	330	YES	6	gustatory receptor 10	*Holotrichia parallela*	49.3	4e-04	34%	AVH87325.1	1.24	2.69
EbraGR10	CL436Contig3	1059	257	NO	5	gustatory receptor 2	*Colaphellus bowringi*	109	8e-24	41%	ALR72528.1	0.51	0.36
EbraGR11	CL16417Contig1	893	254	NO	4	gustatory receptor 16	*Anoplophora chinensis*	130	2e-33	39%	AVN97881.1	0.88	0.89
EbraGR12	CL15196Contig1	586	190	NO	3	gustatory receptor 14	*Pyrrhalta aenescens*	62	8e-08	31%	APC94341.1	0.63	0.79
EbraGR13	EBM_DN24358_c0_g2_i10	1540	174	NO	3	gustatory receptor	*Anoplophora chinensis*	266	1e-80	59%	AUF73061.1	0.09	0.44
EbraGR14	CL436Contig4	4025	141	YES	4	gustatory receptor 2	*Colaphellus bowringi*	43.1	3.1	44%	ALR72528.1	0.14	0.18
EbraGR15	CL20837Contig1	597	110	YES	2	gustatory receptor	*Anopheles darlingi*	43.5	0.16	56%	ETN64058.1	0.77	1.05
EbraGR16	CL20514Contig1	517	108	YES	2	gustatory receptor	*Anoplophora chinensis*	196	5e-58	63%	AUF73061.1	0.21	0.31
EbraGR17	CL1Contig1	764	NO	NO	0	gustatory receptor	*Anoplophora chinensis*	82	3e-14	76%	AUF73061.1	0.58	0.91
EscrSNMP1	ESM_DN23416_c0_g1_i2	4817	408	NO	2	sensory neuron membrane protein SNMP1b	*Colaphellus bowringi*	399	2e-120	62%	ALR72543.1	1.11	1.48
EscrSNMP2	CL15115Contig1	2672	525	YES	2	sensory neuron membrane protein 2	*Dendroctonus ponderosae*	648	0.0	60%	AGI05184.1	7.76	8.25
EscrSNMP3	ESM_DN22450_c0_g1_i5	1618	480	NO	1	sensory neuron membrane protein 1a	*Dendroctonus ponderosae*	741	0.0	79%	AGI05171.1	333.11	342.52
EbraSNMP1	CL39Contig19	5057	533	YES	2	sensory neuron membrane protein SNMP1b	*Colaphellus bowringi*	593	0.0	52%	ALR72543.1	234.87	267.27
EbraSNMP2	CL2304Contig1	2302	525	YES	2	sensory neuron membrane protein 2	*Dendroctonus ponderosae*	624	0.0	60%	AGI05184.1	56.42	51.53
EbraSNMP3	CL6744Contig1	1216	317	NO	1	sensory neuron membrane protein 1a	*Dendroctonus ponderosae*	421	5e-141	74%	AGI05171.1	251.84	272.54

Table S8. Comparison of homologous chemosensory genes in *E. scrobiculatus* and *E. brandti*

Number	Gene name	Gene name	Score	E-value	Identity	Gaps	Number	Gene name	Gene name	Score	E-value	Identity	Gaps
1	EscrOBP1	EbraOBP1	354	5e-132	98%	0%	50	EscrOR1	EbraOR1	395	1e-144	98%	0%
2	EscrOBP2	EbraOBP2	260	1e-94	96%	0%	51	EscrOR2	EbraOR2	734	0.0	92%	0%
3	EscrOBP3	EbraOBP3	304	5e-113	99%	0%	52	EscrOR3	EbraOR3	719	0.0	97%	0%
4	EscrOBP4	EbraOBP4	298	6e-110	98%	0%	53	EscrOR4	EbraOR4	819	0.0	93%	0%
5	EscrOBP5	EbraOBP5	257	4e-95	100%	0%	54	EscrOR5	EbraOR5	676	0.0	91%	0%
6	EscrOBP6	EbraOBP6	294	1e-109	100%	0%	55	EscrOR6	EbraOR6	614	0.0	95%	0%
7	EscrOBP7	EbraOBP7	303	4e-113	100%	0%	56	EscrOR7	EbraOR7	612	0.0	91%	0%
8	EscrOBP8	EbraOBP8	234	2e-86	99%	0%	57	EscrOR8	EbraOR8	663	0.0	91%	0%
9	EscrOBP9	EbraOBP9	305	2e-113	100%	0%	58	EscrOR9	EbraOR9	616	0.0	91%	0%
10	EscrOBP10	EbraOBP10	270	2e-100	100%	0%	59	EscrOR10	EbraOR10	441	8e-162	95%	0%
11	EscrOBP11	EbraOBP11	251	6e-93	94%	0%	60	EscrOR11	EbraOR11	785	0.0	96%	0%
12	EscrOBP12	EbraOBP12	254	6e-94	90%	0%	61	EscrOR12	EbraOR12	748	0.0	94%	0%
13	EscrOBP13	EbraOBP13	259	6e-96	98%	0%	62	EscrOR13	EbraOR13	760	0.0	94%	0%
14	EscrOBP14	EbraOBP14	282	1e-104	93%	0%	63	EscrOR14	EbraOR14	732	0.0	90%	0%
15	EscrOBP15	EbraOBP15	280	4e-96	92%	0%	64	EscrOR15	EbraOR15	764	0.0	91%	0%
16	EscrOBP16	EbraOBP16	270	2e-100	96%	0%	65	EscrOR16	EbraOR16	788	0.0	94%	0%
17	EscrOBP17	EbraOBP17	272	4e-101	99%	0%	66	EscrOR17	EbraOR17	755	0.0	91%	0%
18	EscrOBP18	EbraOBP18	298	1e-110	100%	0%	67	EscrOR18	EbraOR18	780	0.0	94%	0%
19	EscrOBP20	EbraOBP20	238	3e-87	76%	0%	68	EscrOR19	EbraOR19	546	0.0	95%	0%
20	EscrOBP21	EbraOBP21	221	1e-80	83%	0%	69	EscrOR20	EbraOR20	629	0.0	91%	0%
21	EscrOBP23	EbraOBP23	211	4e-77	86%	0%	70	EscrOR21	EbraOR21	775	0.0	91%	0%
22	EscrOBP24	EbraOBP24	97.8	7e-33	86%	0%	71	EscrOR22	EbraOR22	783	0.0	96%	0%
23	EscrOBP25	EbraOBP25	241	5e-89	89%	0%	72	EscrOR23	EbraOR23	767	0.0	95%	0%
24	EscrCSP1	EbraCSP1	252	1e-93	96%	1%	73	EscrOR24	EbraOR24	984	0.0	98%	0%
25	EscrCSP2	EbraCSP2	281	2e-104	98%	0%	74	EscrOR25	EbraOR25	758	0.0	94%	0%
26	EscrCSP3	EbraCSP3	248	3e-91	94%	0%	75	EscrOR26	EbraOR26	704	0.0	85%	0%
27	EscrCSP4	EbraCSP4	263	9e-98	100%	0%	76	EscrOR27	EbraOR27	802	0.0	74%	0%
28	EscrCSP5	EbraCSP5	250	1e-92	95%	0%	77	EscrOR28	EbraOR28	595	0.0	86%	0%
29	EscrCSP6	EbraCSP6	247	2e-91	91%	0%	78	EscrOR29	EbraOR29	613	0.0	88%	0%
30	EscrCSP7	EbraCSP7	248	1e-91	90%	5%	79	EscrOR30	EbraOR30	685	0.0	84%	0%
31	EscrCSP8	EbraCSP8	266	7e-99	93%	0%	80	EscrOR31	EbraOR31	423	9e-155	89%	0%
32	EscrCSP9	EbraCSP9	242	1e-89	97%	0%	81	EscrOR32	EbraOR32	672	0.0	86%	0%
33	EscrCSP10	EbraCSP10	51.2	3e-14	28%	3%	82	EscrOR33	EbraOR33	513	0.0	82%	0%
34	EscrCSP11	EbraCSP11	533	0.0	92%	1%	83	EscrOR34	EbraOR34	741	0.0	88%	0%
35	EscrIR1	EbraIR1	1687	0.0	95%	0%	84	EscrOR35	EbraOR35	715	0.0	88%	0%
36	EscrIR2	EbraIR2	681	0.0	96%	0%	85	EscrOR36	EbraOR36	719	0.0	89%	0%
37	EscrIR3	EbraIR3	1264	0.0	96%	0%	86	EscrOR37	EbraOR37	379	1e-137	87%	0%
38	EscrIR4	EbraIR4	1584	0.0	96%	0%	87	EscrOR38	EbraOR38	721	0.0	87%	0%
39	EscrIR5	EbraIR5	1568	0.0	96%	0%	88	EscrOR39	EbraOR39	362	2e-130	87%	2%
40	EscrIR6	EbraIR6	1882	0.0	97%	0%	89	EscrGR1	EbraGR1	857	0.0	92%	0%
41	EscrIR7	EbraIR7	1171	0.0	92%	1%	90	EscrGR2	EbraGR2	840	0.0	97%	0%
42	EscrIR8	EbraIR8	1463	0.0	90%	6%	91	EscrGR3	EbraGR3	758	0.0	91%	0%
43	EscrIR9	EbraIR9	1754	0.0	98%	0%	92	EscrGR4	EbraGR4	806	0.0	97%	0%
44	EscrIR10	EbraIR10	794	0.0	91%	0%	93	EscrGR5	EbraGR5	449	2e-164	95%	0%
45	EscrIR11	EbraIR11	892	0.0	94%	0%	94	EscrGR6	EbraGR6	795	0.0	88%	0%
46	EscrIR12	EbraIR12	1174	0.0	92%	0%	95	EscrGR10	EbraGR10	409	2e-151	87%	0%
47	EscrIR13	EbraIR13	1169	0.0	95%	0%	96	EscrSNMP1	EbraSNMP1	604	0.0	93%	0%
48	EscrIR14	EbraIR14	885	0.0	98%	0%	97	EscrSNMP2	EbraSNMP2	969	0.0	92%	0%
49	EscrIR15	EbraIR15	1129	0.0	89%	0%	98	EscrSNMP3	EbraSNMP3	602	0.0	95%	0%

Table S9. The primers used for RT-qPCR in *E. scrobiculatus* and *E. brandti*

Name of genes	Forward primer (5'-3')	Reverse primer (3'-5')
EscrOBP1	CGAAACTCAGGCCCTTCAGA	GGGCTTCAAGGGCTTCTGAT
EscrOBP2	AAGGTCGTCACAGAATGCGT	TCGCTTGGTTCTGCCGTATT
EscrOBP3	AGGCGAATTCTCCGACGATC	TTGTCAGCTTGAGGAGGCAG
EscrOBP4	TCGATCGGAAAGCTGCTGAG	TCGAATATTCCCAGGCTGCC
EscrOBP5	GCGATGACCGAAGCACAAAT	ACATGCCGCTCTTGTCTTGA
EscrOBP6	GCAACGCCAGAGGATATCGA	TCCAGCCATTCTGCTTCTGG
EscrOBP7	TATTCCACCGATGGCGAAGG	GGGGTTTCGGGAGGCATTTA
EscrOBP8	GACGATGACGGGGAAATCGA	CGGTCCTTCCCGGTAATAGC
EscrOBP10	CATGGCGTCGAACAAGACAG	TAATGGCCGCCACATTAGCC
EscrOBP11	CAAAGCATGCGTGGGTGAAA	CGTTTGAATTTCACCGGCGT
EscrOBP13	AGCACATTCTCTGCGTCACT	AGGCATTGTAGGCTTGCAGT
EscrOBP14	TTTATCGCCCGACGGAACAA	CCGTCGTCCTCTTGGATCAG
EscrOBP15	GCACGTTGAAGTTGCAGGTT	TCGTCGAACGAGCCATCTTT
EscrOBP16	CTGTCGTAGCTGTCTTGGCT	TGCAAACCGAAATTGGCAGG
EscrOBP17	GAAACACGAATTGACGGGGC	TCTGGTTTCGCCTTCTTGGT
EscrOBP18	GGCGAAATCGATATGAGCGC	CGGATCTGCTTGTTGAACGC
EscrOBP19	TGTGTCAGTGAAAGGTCTGGT	AGGCATTCGGTGATACATTCCA
EscrOBP20	TGATTTCTGCTAGTGCGCTGA	TTCCTTCATGCGTTACCGGT
EscrOBP21	CGTGGGCAAAAAGGAAGGTC	CAGTCTCTTCCGGCGTATCC
EscrOBP22	TTGCGCCAACAAGAAACTGG	GCGAGCAATGTTTGACTGCA
EscrOBP23	GGGGAAGATGACCGATGAGC	GAATCGTCGGAGAAATCGCC
EscrOBP24	CTTGTGGTGGTAAGCGGACT	TTTCGTCCACGCACGTCTTA
EscrOBP26	CGTTCTCCGATAGTCCTGCC	ATCATACGCCGTATCCGCAG
EscrOBP28	GCCGTTCTAGTCTGTGCACT	GTTTGGCATTTTTCGTGCGC
EscrOBP30	TCCGTTGGTCATGTCATCGG	AAACACATGTCTCGCTCCGT
EbraOBP1	CGAAATTCCCACAGCTGCAC	TTCGTTGACGTTCAGGGCTT
EbraOBP2	TTCGCTGCTGCACTCCTTAA	TTCCCCTTGTTCGCCAAACA
EbraOBP3	AGCTGTCGTTTCCGCTATT	CGATAAGGTCCGGGTTTACTT
EbraOBP4	TCTTGTCAAGCCAAGCGTCT	TCCTCTGGTGTAGCCTTGGT
EbraOBP5	GCGATGACCGAAGCACAAAT	ACATGCCGCTCTTGTCTTGA
EbraOBP6	GCAACGCCAGAGGATATCGA	TCCAGCCATTCTGCTTCTGG
EbraOBP8	GCCAGGAAGGGAATTTTCGC	CCCTTTTGAGTGCCGCATTT
EbraOBP9	ATGCATCGGGACGACTTTAG	CATGTCCTGGCAAGCTGATA
EbraOBP10	ACGGCAAGAAAAGCAAAGGT	AAACAATGGCTGGCTTCCCT
EbraOBP12	GGACCTGATCACTCAAGCCC	ATTTCACCGGCGTCGTTTTG
EbraOBP13	AAGGTGCTTTTCGTAGCGGT	GGCCCTTTGTAGGACTGCTT
EbraOBP14	TTCTTATCGCCCGACGGAAC	CGGCATCGTCCTCTTGGATT
EbraOBP15	TCTTCATTGCTGGGTGCTCA	TTGAATTCCTCGTCGTCCCG
EbraOBP16	TGTCCCGGAAACCATCTTCG	CCACGTGTCCTTGCTCATCT
EbraOBP17	TGCTACGAATGTGTTGGCCA	GCCCCGTCAATTCGTGTTTC
EbraOBP18	GTGTAATGTCGGAAAGCGGC	GCTTGTTGAACGCACTGCAA

EbraOBP19	CGAATCCCAAGCCTACCCTG	TCGGCTTTGTGCAGACAGTA
EbraOBP20	GCGGACATGGAAGCTCTCAT	AACGGTGCCATCAGGATTCA
EbraOBP21	GGCAGTGGTCAGTTAGATGCT	CAGTCTCTTCCGGCGTATCC
EbraOBP22	AACTCACGTAGGGCATGTGG	ACGTCCGTGATGTCCTCCTA
EbraOBP23	AACGGCGATTTCTCCGAAGA	TCTAGGTCGTCTCTTTCACCA
EbraOBP24	TAAGACGTGCGTGGTCGAAA	CGCTTGAAGCTCTTCCGGTA
EbraOBP25	GTATGGTGGTAGCAGCCGTT	GCTCTGGCCAGCATATCGTT
EbraOBP26	GGCAGTGGACAGTTAGATGCT	CAGTCTCTTCCGGCGTATCC
EbraOBP28	TACGTAAATCCCCGGGTCCT	CTGCAGCTGCGAGCAATATG
α-tublin	GTTTTGAGCCAGCCAACCAG	AGTAGGACACCAGTCGACGA
rps11	GCTTCAGGGATGTAGAGATTGG	TGCCCTTCGTAACCTTCAAC

Table S10. Protein names and accession numbers used in phylogenetic trees.

OBPs			IRs			ORs		
Number	Protein name	Accession number	Number	Protein name	Accession number	Number	Protein name	Accession number
1	TcasOBP1	EFA05678.1	1	AgamGLURIIa	XP_311343.4	1	AalbOrco	AHL20247.1
2	TcasOBP2	EFA05676.2	2	AgamGLURIIb	XP_311341.5	2	AcorOrco	AKC58535.1
3	TcasOBP3	EFA05675.1	3	AgamGLURIIc	XP_559460.4	3	BmorOR1	NP_001036875.1
4	TcasOBP4	EFA05742.1	4	AgamGLURIId	XP_312117.5	4	BmorOR11	NP_001166604.1
5	TcasOBP05	EFA05677.1	5	AgamGLURIIe	EAA05023.4	5	BmorOR12	NP_001104829.1
6	TcasOBP09	EFA10713.1	6	AgamIR21a	XP_316930.3	6	BmorOR13	NP_001166603.1
7	TcasOBP10	EFA07542.1	7	AgamIR25a	XP_319463.4	7	BmorOR14	NP_001166602.1
8	TcasOBP11	EFA05695.1	8	AgamIR31a	XP_552916.2	8	BmorOR15	NP_001091789.1
9	TcasOBP12	EFA02857.1	9	AgamIR40a	XP_318478.5	9	BmorOR16	NP_001104832.2
10	TcasOBP13	EFA02858.1	10	AgamIR60a	XP_001689273.1	10	BmorOR17	NP_001157210.1
11	TcasOBP14	EFA02914.1	11	AgamIR64a	XP_315013.4	11	BmorOR19	NP_001091785.1
12	TcasOBP15	EFA12066.1	12	AgamIR68a	XP_317522.4	12	BmorOR2	NP_001037060.1
13	TcasOBP16	EFA02853.2	13	AgamIR75d	XP_315067.4	13	BmorOR22	NP_001166613.1
14	TcasOBP17	EFA02861.1	14	AgamIR75l	XP_315470.5	14	BmorOR23	NP_001166606.1
15	TcasOBP18	EFA02860.1	15	AgamIR76b	XP_320564.3	15	BmorOR24	NP_001155300.1
16	TcasOBP19	EFA02960.1	16	AgamIR8a	XP_311538.4	16	BmorOR25	NP_001104828.1
17	TcasOBP20	EFA05793.2	17	AgamNmdar1	EAA00817.3	17	BmorOR27	NP_001166893.1
18	TcasOBP22	EFA09155.2	18	AgamNmdar2	EAA44457.4	18	BmorOR29	NP_001166894.1
19	TcasOBP23	EFA10803.1	19	BmorIR40a	XP_021202684.1	19	BmorOR3	NP_001036925.1
20	TcasOBP24	EFA04576.1	20	BmorIR93a	XP_021203225.1	20	BmorOR30	NP_001091786.1
21	TcasOBP25	EFA04747.2	21	CbowIR2	ALR72541.1	21	BmorOR33	NP_001103623.1
22	TcasOBP26	EFA04746.2	22	CbowIR41a	ALR72539.1	22	BmorOR35	NP_001103476.1
23	TcasOBP-C01	EFA07544.1	23	CbowIR5	ALR72540.1	23	BmorOR36	NP_001166892.1
24	TcasOBP-C02	EFA07545.1	24	CbowIR6	ALR72535.1	24	BmorOR4	NP_001036926.1
25	TcasOBP-C03	EFA07546.1	25	CbowIR75q	ALR72537.1	25	BmorOR40	NP_001166608.1
26	TcasOBP-C04	EFA07430.1	26	CbowIR8a	ALR72538.1	26	BmorOR41	NP_001091787.1
27	TcasOBP-C05	EFA07543.1	27	DmelGluRIIA	NP_523484.2	27	BmorOR45	NP_001104798.1
28	TcasOBP-C09	EFA07429.1	28	DmelGluRIIB	NP_523485.3	28	BmorOR47	NP_001104818.1
29	TcasOBP-C10	EEZ97741.1	29	DmelGluRIIC	NP_608557.4	29	BmorOR5	NP_001036927.1
30	TcasOBP-C11	EEZ97739.1	30	DmelGluRIID	NP_651982.1	30	BmorOR54	NP_001166616.1
31	TcasOBP-C12	EEZ97740.1	31	DmelGluRIIE	NP_001036733.1	31	BmorOR55	NP_001166612.1
32	TcasOBP-C13	EEZ97789.1	32	DmelIR100a	NP_651898.2	32	BmorOR56	NP_001166617.1
33	TcasOBP-C14	EFA07518.1	33	DmelIR10a	NP_001096949.1	33	BmorOR59	NP_001166611.1
34	TcasOBP-C15	EFA02826.1	34	DmelIR11a	NP_572795.2	34	BmorOR61	NP_001166619.1
35	TcasOBP-C16	EFA02889.1	35	DmelIR20a	NP_608456.1	35	BmorOR63	NP_001166620.1
36	TcasOBP-C17	EFA02890.1	36	DmelIR21a	NP_001097043.1	36	BmorOR64	NP_001166621.1
37	TcasOBP-C18	EEZ99197.1	37	DmelIR25a	NP_001260049.1	37	BmorOR7	NP_001106227.1
38	DponOBP1	AKK25129.1	38	DmelIR31a	NP_001260346.1	38	BmorOR8	NP_001157209.1
39	DponOBP2	AKK25130.1	39	DmelIR40a	NP_610140.4	39	BmorOR9	NP_001116805.1
40	DponOBP3	AKK25131.1	40	DmelIR41a	NP_995744.4	40	CbowOR1	ALR72546.1
41	DponOBP5	AKK25133.1	41	DmelIR47a	NP_610580.1	41	CbowOR10	ALR72555.1
42	DponOBP6	AKK25134.1	42	DmelIR48b	NP_610697.1	42	CbowOR11	ALR72556.1
43	DponOBP10	AKK25136.1	43	DmelIR48c	NP_610700.1	43	CbowOR13	ALR72558.1
44	DponOBP12	AKK25137.1	44	DmelIR51b	NP_725440.1	44	CbowOR14	ALR72559.1
45	DponOBP13	AKK25138.1	45	DmelIR52a	NP_611041.2	45	CbowOR15	ALR72560.1
46	DponOBP15	AKK25139.1	46	DmelIR52b	NP_725469.2	46	CbowOR17	ALR72562.1
47	DponOBP16	AKK25140.1	47	DmelIR52c	NP_725470.1	47	CbowOR19	ALR72564.1
48	DponOBP17	AKK25141.1	48	DmelIR52d	NP_611042.2	48	CbowOR20	ALR72565.1
49	DponOBP18	AKK25142.1	49	DmelIR54a	NP_611259.2	49	CbowOR21	ALR72566.1
50	DponOBP19	AKK25143.1	50	DmelIR56a	NP_725850.1	50	CbowOR22	ALR72567.1
51	DponOBP20	AKK25144.1	51	DmelIR56b	NP_611430.1	51	CbowOR24	ALR72568.1
52	Dmellush-A	NP_524162.1	52	DmelIR56c	NP_611431.1	52	CbowOR26	ALR72569.1
53	DmelOBP18a	NP_573350.1	53	DmelIR56d	NP_611432.1	53	CbowOR27	ALR72570.1
54	DmelOBP19a	NP_728338.2	54	DmelIR60a	NP_611901.1	54	CbowOR28	ALR72571.1
55	DmelOBP19b	NP_608391.2	55	DmelIR60b	NP_001137755.1	55	CbowOR29	ALR72572.1
56	DmelOBP19c-A	NP_608392.1	56	DmelIR60d	NP_001137757.2	56	CbowOR3	ALR72548.1
57	DmelOBP19d-A	NP_523421.2	57	DmelIR60e	NP_611927.3	57	CbowOR31	ALR72574.1
58	DmelOBP19d-B	NP_788940.1	58	DmelIR62a	NP_001033986.1	58	CbowOR32	ALR72575.1
59	DmelOBP22a-B	NP_001014457.1	59	DmelIR64a	NP_647962.1	59	CbowOR34	ALR72577.1
60	DmelOBP22a-C	NP_722746.2	60	DmelIR67a	NP_648329.3	60	CbowOR35	ALR72578.1

61	DmelOBP28a	NP_523505.1	61	DmelIR67b	NP_648393.1	61	CbowOR36	ALR72579.1
62	DmelOBP44a-A	NP_610358.1	62	DmelIR67c	NP_729609.1	62	CbowOR37	ALR72580.1
63	DmelOBP46a	NP_610574.1	63	DmelIR68a	NP_648455.2	63	CbowOR38	ALR72581.1
64	DmelOBP47a-A	NP_610632.1	64	DmelIR68b	NP_648548.1	64	CbowOR4	ALR72549.1
65	DmelOBP47a-B	NP_995810.1	65	DmelIR75a	NP_649012.2	65	CbowOR40	ALR72583.1
66	DmelOBP47b	NP_610669.1	66	DmelIR75b	NP_001137966.2	66	CbowOR5	ALR72550.1
67	DmelOBP49a	NP_610812.1	67	DmelIR75c	NP_649013.3	67	CbowOR6	ALR72551.1
68	DmelOBP50a-B	NP_995832.1	68	DmelIR75d	NP_649074.2	68	CbowOR7	ALR72552.1
69	DmelOBP50a-A	NP_725385.1	69	DmelIR76b	NP_649176.1	69	CbowOR9	ALR72554.1
70	DmelOBP50e	NP_610959.2	70	DmelIR7a	NP_572406.1	70	CbowOrco	ALR72547.1
71	DmelOBP51a	NP_725436.1	71	DmelIR7b	NP_572410.2	71	DmelOR22a	NP_523453.1
72	DmelOBP56a	NP_611442.1	72	DmelIR7d	NP_001138175.1	72	DmelOR22b	NP_477425.1
73	DmelOBP56b	NP_611443.1	73	DmelIR7e	NP_001138176.1	73	DmelOR23a	NP_523458.3
74	DmelOBP56c-B	NP_995902.1	74	DmelIR7f	NP_001138177.1	74	DmelOR24a	NP_523470.3
75	DmelOBP56c-C	NP_725925.3	75	DmelIR7g	NP_572413.2	75	DmelOR33a	NP_523553.1
76	DmelOBP56d-A	NP_611444.2	76	DmelIR84a	NP_649720.2	76	DmelOR33b	NP_523554.1
77	DmelOBP56e-A	NP_611445.1	77	DmelIR85a	NP_649833.1	77	DmelOR33c	NP_523555.1
78	DmelOBP56e-B	NP_001286620.1	78	DmelIR87a	NP_650290.2	78	DmelOR43a	NP_523647.2
79	DmelOBP56f	NP_725926.1	79	DmelIR8a	NP_727328.1	79	DmelOR45a	NP_523666.3
80	DmelOBP56g-A	NP_611447.1	80	DmelIR92a	NP_001097845.2	80	DmelOR47a	NP_523689.1
81	DmelOBP56g-B	NP_995903.1	81	DmelIR93a	NP_650924.3	81	DmelOR47b	NP_523690.3
82	DmelOBP56h-A	NP_611448.2	82	DmelIR94a	NP_732699.1	82	DmelOR49a	NP_523711.3
83	DmelOBP57a	NP_725966.1	83	DmelIR94b	NP_732700.2	83	DmelOR56a	NP_523796.2
84	DmelOBP57b-A	NP_725965.1	84	DmelIR94c	NP_732701.2	84	DmelOR59a	NP_523821.1
85	DmelOBP57c	NP_611481.1	85	DmelIR94d	NP_001138099.1	85	DmelOR59b	NP_523822.1
86	DmelOBP57d	NP_725973.1	86	DmelIR94e	NP_001097885.2	86	DmelOR59c	NP_523823.1
87	DmelOBP57e	NP_611488.1	87	DmelIR94f	NP_732868.2	87	DmelOR65a	NP_729161.1
88	DmelOBP58b	NP_611709.1	88	DmelIR94g	NP_651147.2	88	DmelOR65b	NP_729162.3
89	DmelOBP58c	NP_611710.1	89	DmelIR94h	NP_651148.2	89	DmelOR65c	NP_729163.2
90	DmelOBP58d	NP_611711.1	90	DmelNmdar1	NP_730940.1	90	DmelOR67a	NP_524005.2
91	DmelOBP59a	NP_788429.1	91	DmelNmdar2	NP_001162636.1	91	DmelOR67b	NP_524007.2
92	DmelOBP83a-A	NP_524241.1	92	DponIR25a	JAA74471.1	92	DmelOR67c	NP_524018.2
93	DmelOBP83a-C	NP_001287190.1	93	DponIR75q	JAA74481.1	93	DmelOR7a	NP_511081.1
94	DmelOBP83b	NP_524242.2	94	DponIR76b	JAA74508.1	94	DmelOR83a	NP_524234.2
95	DmelOBP83ef	NP_731042.1	95	DponIR8a	AGI05169.1	95	DmelOR83b	CBA13843.1
96	DmelOBP93a	NP_650945.1	96	DponIR93a.1	JAA74495.1	96	DmelOR83c	NP_524244.2
97	DmelOBP99a-A	NP_651707.1	97	DponIR41a	JAA74477.1	97	DmelOR85b	NP_524279.2
98	DmelOBP99b-A	NP_651713.1	98	DponnIR75p.1	JAA74497.1	98	DmelOR85c	NP_524280.2
99	DmelOBP99b-B	NP_001263078.1	99	OtauIR21a	XP_022915376.1	99	DmelOR88a	NP_524348.2
100	DmelOBP99d	NP_651712.1	100	OtauIR25a	XP_022900502.1	100	DmelOR92a	NP_524414.2
101	RferOBP1	ANE37545.1	101	OtauIR93a-X2	XP_022920713.1	101	DmelOR94a	NP_524455.1
102	RferOBP2	ANE37546.1	102	TmolIR1	AJO62239.1	102	DmelOR98a	NP_524536.2
103	RferOBP3	ANE37547.1	103	TmolIR2	AJO62240.1	103	DmelOrco	NP_001097687.1
104	RferOBP4	ANE37548.1	104	TmolIR4	AJO62242.1	104	DponOR11	JAA74516.1
105	RferOBP5	ANE37549.1	105	TmolIR5	AJO62243.1	105	DponOR12	JAA74465.1
106	RferOBP6	ANE37550.1	106	TmolIR6	AJO62244.1	106	DponOR15	JAA74461.1
107	RferOBP7	ANE37551.1		**GRs**		107	DponOR16	JAA74512.1
108	RferOBP8	ANE37552.1	Number	Protein name	Accession number	108	DponOR19	JAA74501.1
109	RferOBP9	ANE37553.1	1	DmelGR10a	NP_727523.1	109	DponOR24	JAA74513.1
110	RferOBP10	ANE37554.1	2	DmelGR10b	NP_511121.1	110	DponOR25	JAA74518.1
111	LoryOBP1	AHE13800.1	3	DmelGR21aB	NP_001259841.1	111	DponOR3	JAA74476.1
112	LoryOBP2	AHE13799.1	4	DmelGR22a	NP_722733.1	112	DponOR30	JAA74510.1
113	LoryOBP3	AHE13791.1	5	DmelGR22b	NP_001014456.1	113	DponOR32	JAA74463.1
114	LoryOBP5	AHE13798.1	6	DmelGR22c	NP_722732.2	114	DponOR33	JAA74484.1
115	LoryOBP6	AHE13797.1	7	DmelGR22e	NP_722731.1	115	DponOR39	JAA74479.1
116	LoryOBP11	AHE13795.1	8	DmelGR22f	NP_722729.2	116	DponOR40	JAA74491.1
117	LoryOBP12	AHE13794.1	9	DmelGR28a	NP_523504.2	117	DponOR45	JAA74506.1
118	LoryOBP14	AHE13793.1	10	DmelGR28bA	NP_647614.2	118	DponOR5	JAA74473.1
119	LoryOBP16	AHE13792.1	11	DmelGR28bB	NP_995643.1	119	DponOR6	JAA74487.1
120	AglaPBP1	ASA46120.1	12	DmelGR28bC	NP_995642.1	120	DponOR7	JAA74504.1
121	AglaPBP2	ASA46121.1	13	DmelGR28bD	NP_995641.1	121	DponOrco	XP_019768125.1
122	BhorPBP1	AIV43008.1	14	DmelGR28bE	NP_995640.1	122	ItypOR1	JAA74440.1
123	BhorPBP2	AIV43009.1	15	DmelGR32a	NP_523543.3	123	ItypOR11	JAA74439.1

124	HparPBP1	ADF87391.1
125	AosaPBP	AAC63437.1
126	HelePBP	AGM37951.1
127	PjapPBP	AAC63436.1
128	ArufPBP	BAF79995.1
129	AoctPBP1	BAC06499.1
130	AschPBP	BAF79601.1
131	CbowOBP1	ALR72489.1
132	CbowOBP2	ALR72490.1
133	CbowOBP4	ALR72492.1
134	CbowOBP5	ALR72493.1
135	CbowOBP7	ALR72495.1
136	CbowOBP8	ALR72496.1
137	CbowOBP9	ALR72497.1
138	CbowOBP10	ALR72498.1
139	CbowOBP12	ALR72500.1
140	CbowOBP13	ALR72501.1
141	CbowOBP14	ALR72502.1
142	CbowOBP15	ALR72503.1
143	CbowOBP17	ALR72505.1
144	CbowOBP18	ALR72506.1
145	CbowOBP19	ALR72507.1
146	CbowOBP20	ALR72508.1
147	CbowOBP22	ALR72510.1
148	CbowOBP23	ALR72511.1
149	CbowOBP26	ALR72514.1

CSPs

Number	Protein name	Accession number
1	BmorCSP1	AAM34276.1
2	BmorCSP2	AAM34275.1
3	BmorCSP3	ABH88196.1
4	BmorCSP4	ABH88197.1
5	BmorCSP5	ABH88198.1
6	BmorCSP6	ABH88199.1
7	BmorCSP7	ABH88200.1
8	BmorCSP8	ABH88201.1
9	BmorCSP9	ABH88202.1
10	BmorCSP10	ABH88203.1
11	BmorCSP11	ABH88204.1
12	BmorCSP12	ABH88205.1
13	BmorCSP13	ABH88206.1
14	BmorCSP14	ABH88207.1
15	BmorCSP15	ABH88208.1
16	BmorCSP16	ABH88209.1
17	ItypCSP1	JAA74387.1
18	ItypCSP4	JAA74385.1
19	ItypCSP5	JAA74384.1
20	TmolCSP1	AJO62207.1
21	TmolCSP2	AJO62208.1
22	TmolCSP3	AJO62209.1
23	TmolCSP4	AJO62210.1
24	TmolCSP5	AJO62211.1
25	TmolCSP6	AJO62212.1
26	TmolCSP7	AJO62213.1
27	TmolCSP8	AJO62214.1
28	TmolCSP9	AJO62215.1
29	TmolCSP10	AJO62216.1
30	TmolCSP11	AJO62217.1
31	TmolCSP12	AJO62218.1
32	LoryCSP3	AHE13801.1
33	LoryCSP6	AHE13802.1
34	LoryCSP8	AHE13803.1
35	LoryCSP9	AHE13804.1

16	DmelGR39b	NP_724336.1
17	DmelGR43aA	NP_523650.2
18	DmelGR43aB	NP_001036531.1
19	DmelGR43aC	NP_001286158.1
20	DmelGR47b	NP_725040.2
21	DmelGR57a	NP_523798.1
22	DmelGR58b	NP_523808.2
23	DmelGR58c	NP_726133.2
24	DmelGR59a	NP_726287.2
25	DmelGR59c	NP_726292.1
26	DmelGR59d	NP_611758.2
27	DmelGR5a	NP_511050.1
28	DmelGR61aB	AGB93921.1
29	DmelGR63a	NP_001137883.1
30	DmelGR64a	NP_728920.1
31	DmelGR64b	NP_728921.1
32	DmelGR64cA	AAF47824.2
33	DmelGR64dC	NP_001027105.2
34	DmelGR64eA	NP_728923.2
35	DmelGR64f	NP_728924.2
36	DmelGR68a	NP_524027.2
37	DmelGR89a	NP_650555.2
38	DmelGR92a	NP_732489.2
39	DmelGR93b	NP_732664.2
40	DmelGR93c	NP_732665.1
41	DmelGR94a	NP_732816.1
42	DmelGR98b	NP_733213.1
43	DponGR3	JAA74468.1
44	ItypGR6	JAA74417.1
45	ItypGR3	JAA74415.1
46	OtauGR39a	XP_022910663.1
47	OtauGR64f	XP_022908857.1
48	OtauGR63a	XP_022904812.1
49	OtauGR66a	XP_022912957.1
50	TcasGR20	EFA05758.1
51	TcasGR21	EFA13587.1
52	TcasGR23	EFA10796.1
53	TcasGR28	EFA05761.1
54	TcasGR30	EFA01379.2
55	TcasGR37	EFA09283.1
56	TcasGR43	EFA05768.1
57	TcasGR46	EFA05771.1
58	TcasGR48	EFA05773.1
59	TcasGR49	EFA05774.1
60	TcasGR52	EFA05777.2
61	TcasGR64e	KYB27391.1
62	TcasGR66	NP_001138955.1
63	TcasGR67	EFA05778.1
64	TcasGR68	EFA05779.2
65	TcasGR72	EFA11815.1
66	TcasGR76	EFA09285.1
67	TcasGR87	EEZ99384.1
68	TcasGR89	EFA12700.1
69	TcasGR91	EFA12699.1
70	TcasGR92	EFA12176.1
71	TcasGR97	EFA02931.1
72	TcasGR98	EFA02932.2
73	TcasGR109	NP_001138957.1
74	TcasGR117	EFA07614.1
75	TcasGR118	EFA07615.1
76	TcasGR119	EFA07616.1
77	TcasGR120	EFA07617.1
78	TcasGR121	EFA07618.1

124	ItypOR12	JAA74435.1
125	ItypOR13	JAA74448.1
126	ItypOR16	JAA74455.1
127	ItypOR18	JAA74450.1
128	ItypOR19	JAA74446.1
129	ItypOR2	JAA74453.1
130	ItypOR23	JAA74456.1
131	ItypOR27	JAA74445.1
132	ItypOR28	JAA74459.1
133	ItypOR3	JAA74437.1
134	ItypOR31	JAA74457.1
135	ItypOR32	JAA74458.1
136	ItypOR34	JAA74454.1
137	ItypOR35	JAA74441.1
138	ItypOR36	JAA74443.1
139	ItypOR39	JAA74436.1
140	ItypOR4	JAA74449.1
141	ItypOR6	JAA74442.1
142	ItypOR7	JAA74438.1
143	ItypOR9	JAA74447.1
144	TcasOR1	EFA05687.1
145	TcasOR14	EFA09245.2
146	TcasOR15	EFA09246.1
147	TcasOR154	EEZ97777.1
148	TcasOR16	EFA09170.1
149	TcasOR160	EFA02947.1
150	TcasOR165	EFA02949.1
151	TcasOR17	EFA01293.1
152	TcasOR198	EFA07457.1
153	TcasOR202	EEZ97783.1
154	TcasOR208	EFA01396.1
155	TcasOR217	EFA01402.1
156	TcasOR222	EFA01407.1
157	TcasOR223	EFA01408.1
158	TcasOR229	EFA09267.1
159	TcasOR23	EFA10798.1
160	TcasOR24	EFA10799.2
161	TcasOR25	EFA01335.1
162	TcasOR26	EEZ99239.1
163	TcasOR263	EEZ99422.1
164	TcasOR30	EEZ99405.1
165	TcasOR34	EEZ99230.1
166	TcasOR40	EEZ99410.2
167	TcasOR47	EFA02940.1
168	TcasOR48	EEZ99304.2
169	TcasOR49	EEZ99303.1
170	TcasOR50	EEZ99413.2
171	TcasOR51	EEZ99302.1
172	TcasOR52	EEZ99301.1
173	TcasOR53	EFA01295.2
174	TcasOR54	EEZ97743.1
175	TcasOR57	EFA05727.1
176	TcasOR60	EEZ99415.2
177	TcasOR67	EEZ97776.1
178	TcasOR68	EEZ99310.2
179	TcasOR76	EFA10779.1
180	TcasOR77	EFA10701.1
181	TmolOrco	AJO62219.1

SNMPs

Number	Protein name	Accession number
1	DmelSNMP1	NP_001262803.1
2	DmelSNMP2	NP_001261539.1
3	DponSNMP1a	AGI05171.1

36	LoryCSP10	AHE13805.1
37	MaltCSP1	AIX97041.1
38	MaltCSP2	AIX97042.1
39	MaltCSP3	AIX97043.1
40	MaltCSP4	AIX97044.1
41	MaltCSP5	AIX97045.1
42	MaltCSP6	AIX97046.1
43	MaltCSP7	AIX97047.1
44	MaltCSP8	AIX97040.1
45	MaltCSP9	AIX97084.1
46	MaltCSP10	AIX97085.1
47	MaltCSP11	AIX97086.1
48	MaltCSP12	AIX97087.1
49	DponCSP1	AGI05161.1
50	DponCSP2	AGI05172.1
51	DponCSP3	AGI05160.1
52	DponCSP4	AKK25148.1
53	DponCSP6	AGI05162.1
54	DponCSP8	AGI05164.1
55	DponCSP11	AGI05163.1
56	TcasCSP1	EFA07423.1
57	TcasCSP2	EFA07420.1
58	TcasCSP3	EFA07417.1
59	TcasCSP4	EFA07418.1
60	TcasCSP5	EFA07419.1
61	TcasCSP6	EFA07421.1
62	TcasCSP7	EFA07424.1
63	TcasCSP9	EFA07422.1
64	TcasCSP10	EFA07552.1
65	TcasCSP11	EFA07563.1
66	TcasCSP12	EFA07566.1
67	TcasCSP16	EFA07567.1
68	TcasCSP17	EEZ99322.1
69	TcasCSP18	EFA07570.1
70	TcasCSP19	EFA07577.1
71	TcasCSP20	EFA01297.1

79	TcasGR125	EFA07621.2
80	TcasGR128	EFA07622.1
81	TcasGR131	EFA07624.1
82	TcasGR133	EFA07626.1
83	TcasGR156	EFA09288.1
84	TcasGR160	EFA12223.1
85	TcasGR164	EEZ99392.1
86	TcasGR204	EFA07637.1

4	DponSNMP2	AGI05184.1
5	ItypSNMP1	JAA74404.1
6	ItypSNMP2	JAA74403.1
7	CbowSNMP1b	ALR72543.1
8	CbowSNMP2	ALR72544.1
9	TmolSNMP1	AJO62245.1
10	TmolSNMP2	AJO62246.1
11	TcasSNMP1	EFA02899.2
